# Tobacco, Alcohol, Cannabis, and Other Drug Use in the US Before and During the Early Phase of the COVID-19 Pandemic

**DOI:** 10.1001/jamanetworkopen.2022.54566

**Published:** 2023-01-31

**Authors:** Wilson M. Compton, Kerry S. J. Flannagan, Marushka L. Silveira, MeLisa R. Creamer, Heather L. Kimmel, Moana Kanel, Carlos Blanco, Nora D. Volkow

**Affiliations:** 1 National Institute on Drug Abuse, National Institutes of Health, Bethesda, Maryland; 2 Kelly Government Solutions, Rockville, Maryland

## Abstract

**Importance:**

Information about national substance use trends among youths and adults after mid-March 2020 is limited due to constraints on surveillance during the COVID-19 pandemic.

**Objective:**

To evaluate whether substance use prevalence in the early part of the pandemic (2020) differed from the prepandemic periods of 2018 to 2019 and 2016 to 2018.

**Design, Setting, and Participants:**

This cross-sectional study was a repeated analysis of 2016 to 2020 data from a nationally representative sample of youths and adults in the Population Assessment of Tobacco and Health (PATH) Study. Participants were representative of the US civilian noninstitutionalized population. Household residents age 13 years or older were interviewed in person from 2016 to 2019 and via telephone in 2020.

**Exposures:**

Age, calendar year.

**Main Outcomes and Measures:**

Past 30-day self-reported use of any tobacco, any alcohol, binge drinking, cannabis, and any other illegal or misused prescription drugs.

**Results:**

The overall nationally representative 2020 sample included 7129 youths (ages 13-17 years), 3628 young adults (ages 18-20 years), and 8874 adults (ages ≥21 years). Comparing 2018 to 2019 with 2020 among youths, prevalence of all substances used declined (eg, cannabis use declined in those aged 16-17 years from 14.9% to 7.6%; absolute difference, −7.3 percentage points [95% CI −8.8 to −5.8 percentage points]). Among young adults, prevalence of all substances other than any alcohol decreased significantly (eg, tobacco use declined from 37.8% to 22.8%; absolute difference, −15.1 percentage points [95% CI −16.8 to −13.3 percentage points]). In adults ages 21 to 24 years, any tobacco use declined from 39.0% to 30.9% (absolute difference, −8.2 percentage points [95% CI, −10.6 to −5.7 percentage points]), and alcohol use increased from 60.2% to 65.2% (absolute difference, 5.0 percentage points [95% CI, 2.3 to 7.7 percentage points]). Among adults aged 25 years or older, any tobacco use declined from 39.0% to 30.9% (absolute difference, −8.2 percentage points [95% CI, −10.6 to −5.7 percentage points]), cannabis use increased from 11.3% to 12.4% (absolute difference, 1.2 percentage points [95% CI, 0.3 to 2.0 percentage points]), and other substance use declined from 5.8% to 3.7% (absolute difference, −2.1 percentage points [95% CI, −2.9 to −1.4 percentage points]).

**Conclusions and Relevance:**

In this cross-sectional study, substance use decreased between 2019 and 2020 among those aged 13 to 20 years; consistent declines were not seen in older persons other than tobacco use reductions, and cannabis use increased among adults ages 25 years and older. While social changes during the COVID-19 pandemic could have affected substance use, findings should be interpreted with caution due to differences in data collection methods in 2016 to 2019 and 2020.

## Key Points

**Question:**

How do rates of substance use in the US during the COVID-19 pandemic in 2020 compare with earlier years?

**Findings:**

In this cross-sectional study of 19 631 youths, young adults, and adults, substance use substantially decreased across nearly all substance categories in 2018 to 2019 vs 2020 among youths and young adults, whereas consistent changes were not seen in older persons.

**Meaning:**

While differences in data collection methods may have contributed to observed changes, these findings suggest that despite significant stressors during 2020, substance use declined in youths; reductions in youths but not adults during this period of social isolation may reflect, in part, youth-specific sensitivity to peer influences on substance use.

## Teaser

This cross-sectional study evaluates whether substance use prevalence in the early phase of the COVID-19 pandemic differed from the prepandemic periods of 2018 to 2019 and 2016 to 2018 among US residents aged 13 years or older.

## Introduction

Responses to the spread of the SARS-CoV-2 virus in the US included massive shifts to social functioning. Schools were closed. Public gatherings were severely curtailed. Travel and social interactions with persons outside of a household were markedly reduced. In addition to these well-known changes, the collection of much scientific and public health data was also stopped abruptly in March 2020. These abrupt stoppages included discontinuation of the annual Monitoring the Future Study of secondary students and discontinuation of the National Survey on Drug Use and Health. Monitoring the Future collects data from hundreds of schools across the US, and with the closure of most schools, its data collection ceased in March 2020.^1^ Similarly, the National Survey on Drug Use and Health includes in-person interviews conducted in households across all 50 states (and the District of Columbia) which were not possible in the face of the COVID-19 public health emergency.^2^ Along with the Youth Risk Behavior Survey,^3^ which was not scheduled for data collection in 2020, Monitoring the Future and the National Survey on Drug Use and Health are the major sources of data on drug use trends in the US general population, and so their discontinuation, although temporary, created a major gap in information that is relied on by public health officials, policy makers, and practitioners.

In response to these markedly constrained surveillance activities during the early months of the COVID-19 pandemic, new sources of data have been sought, and in response, we report data drawn from a large, existing cohort of participants in the Population Assessment of Tobacco and Health (PATH) Study.^3,4,5^ While the PATH Study had been conducted for 7 prior years using in-person interviewing, during the pandemic the PATH Study shifted to telephone administration of interviews for the sample that was already established. Using this novel source of information, we aimed to evaluate whether prevalence of substance use in the last half of 2020 differed from earlier years for youths in their teens and adults. While the PATH Study does not have the same number of detailed measures of substance use as the National Survey on Drug Use and Health or the Monitoring the Future Study, it has consistently collected self-report information about the full range of tobacco products as well as self-report regarding use of alcohol, cannabis, and other substances since 2013. Thus, the PATH Study provided a way to determine whether substance use prevalence in the latter half of 2020 differed from 2018 to 2019 and 2016 to 2018 among youths (ages 13-17 years), young adults (ages 18-20 and 21-24 years), and older adults (ages 25 years and older).

## Methods

The PATH Study is an ongoing, nationally representative, longitudinal study of US youths (ages 12-17 years) and adults (ages ≥18 years). The PATH Study data collection is conducted by Westat, and these procedures have been approved by Westat’s institutional review board. Adult respondents provided written informed consent; youth respondents provided written assent with parent (or legal guardian) written consent. This article follows the Strengthening the Reporting of Observational Studies in Epidemiology (STROBE) reporting guideline.

The PATH Study used a stratified address-based, area-probability sampling design allowing the study of a nationally representative longitudinal sample of youths and adults in the US that oversampled tobacco users, those aged 18 to 24 years, and African American individuals. For this analysis, PATH Study data were collected from participants aged 13 years and older using audio computer-assisted self-interviews conducted in English or Spanish. While data on 12-year-olds were available for the years prior to 2020, because of the inability to recruit 12-year-olds in 2020, data were limited to those 13 years of age and older (ie, already existing participants). All previous waves of data were limited to the same age range of 13 years and older as the 2020 data to assure comparability.

Pre–COVID-19 data were from December 1, 2016, through January 3, 2018 (wave 4 [W4]), from December 1, 2017, through December 1, 2018, for ages 13 to 17 years only (W4.5), and from December 1, 2018, through November 30, 2019, for ages 13 and older (W5) (eFigure in Supplement 1). For data from the early months of the COVID-19 pandemic, the study questionnaire was adapted for telephone administration to participants aged 13 to 19 years during July 3 through December 31, 2020, and was similarly adapted for telephone administration in the PATH Study Adult Telephone Survey (PATH-ATS) from September 10 through December 20, 2020, among a subsample aged 20 years and older.

Further details regarding the PATH Study design and methods^4,5,6^ and demographic and tobacco use distributions^7^ are published elsewhere. Details on interviewing procedures, questionnaires, sampling, weighting, response rates, and accessing PATH Study Restricted Use Files are also available.^8^

### Measures

Substance use measures were past 30-day self-reported any tobacco (cigarettes, electronic tobacco products [e-products], traditional cigars, cigarillos, filtered cigars, hookah, pipe, smokeless tobacco, or snus in either loose or pouched form [for youths, also dissolvable tobacco, bidis, or kreteks]), any alcohol, binge drinking, cannabis, and any other illegal or misused prescription drugs. eTable 1 in Supplement 1 provides definitions.

Covariates included age (13-15, 16-17, 18-20, 21-24, and ≥25 years), sex, race and ethnicity (Hispanic, non-Hispanic White, non-Hispanic Black, and non-Hispanic other [ie, American Indian or Alaska Native, Asian Indian, Chinese, Filipino, Japanese, Korean, Vietnamese, other Asian, Native Hawaiian, Guamanian or Chamorro, Samoan, other Pacific Islander, and multiracial]), annual household income (<$50 000 and ≥$50 000), and solely for ages 18 to 24 years, enrolled vs nonenrolled in technical or vocational, 2-year, 4-year, graduate or professional, and other postsecondary degree programs. Multiple studies have documented variation in substance use among persons with differing racial and ethnic backgrounds. Therefore, we adjusted for race and ethnicity to isolate associations with age as much as possible. These covariates were determined from self-report information in response to PATH Study questions. Specifically, race and ethnicity were determined according to PATH Study respondents’ self-classification of racial and ethnic origin and identification based on classifications developed by the US Census Bureau.

### Statistical Analysis

To adjust for complex sample design and nonresponse,^5^ all prevalence estimates were weighted, using W4 cohort cross-sectional or pseudo–cross-sectional weights as appropriate to produce nationally representative estimates and account for nonresponse and loss to follow up; 95% CIs were computed using balanced repeated replication methods^9^ with the Fay adjustment set to 0.3 to increase estimate stability.^10^ Because the rate of missing data was low (≤1% missing for all substance use variables), we conducted a complete-case analysis. Estimates were flagged for low statistical precision if based on a denominator of fewer than 50 or if the coefficient of variation of the estimate or its complement was larger than 30%.^11^ *P* values were used to compare prevalence of each substance at each prior wave to 2020 using Rao-Scott χ^2^ tests. Because the focus of this analysis was on describing substance use trends over time, statistical testing was not of primary interest, and we did not adjust for multiple comparisons.

In supplemental analyses, estimates were stratified by age and either sex, race and ethnicity, annual household income, or postsecondary degree enrollment (age 18-24 years only). *P* values for interactions were estimated using logistic regression models with substance use as outcomes and with factors including main terms for year and the second stratification variable. *P* values of interaction terms were from interaction *F* tests that compared the data from 2018 to 2019 with 2020 for prevalence differences between these 2 data collection waves. Data analyses were conducted with SAS version 9.4 (SAS Institute). Statistical significance was prespecified at *P* < .05 with 2-tailed tests. Data were analyzed between January 2022 and July 2022.

## Results

**Figure 1.**
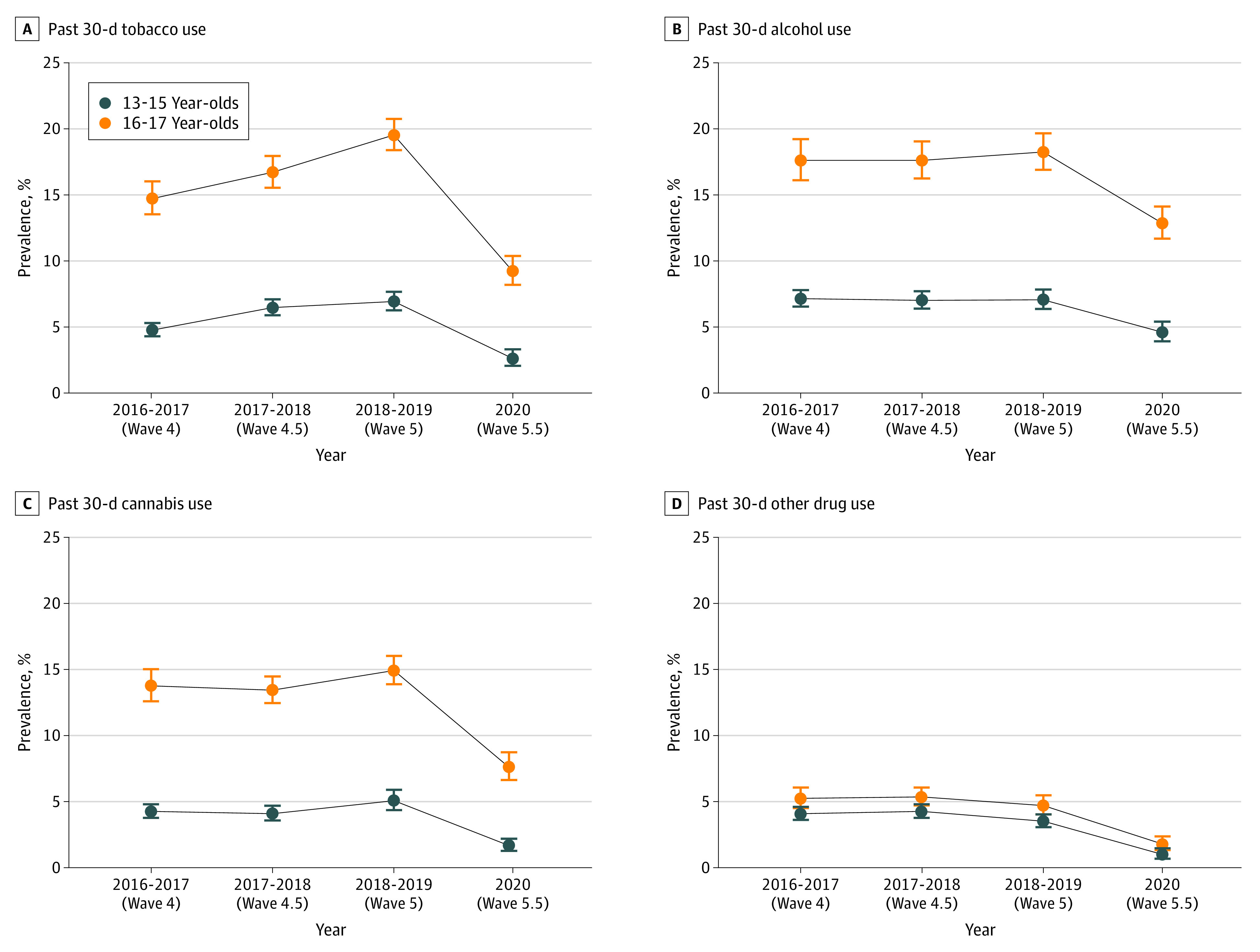
Past 30-Day Tobacco, Alcohol, Cannabis, and Other Drug Use Among Those Aged 13 to 15 and 16 to 17 Years in the Population Assessment of Tobacco and Health Study, 2016 to 2017 vs 2020

**Figure 2.**
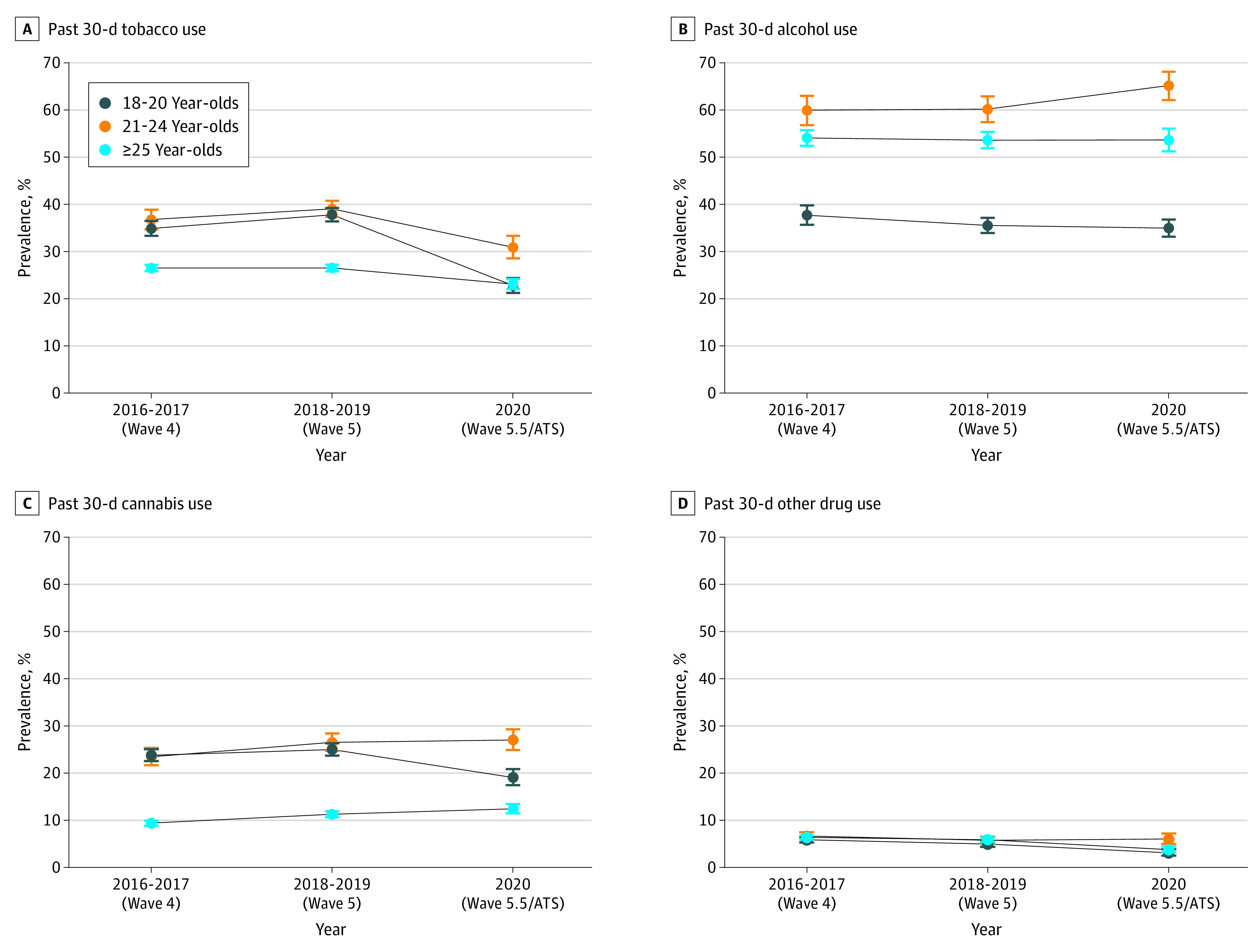
Past 30-Day Tobacco, Alcohol, Cannabis, and Other Drug Use Among Those Aged 18 to 20, 21 to 24, and 25 Years or Older in the Population Assessment of Tobacco and Health Study, 2016 to 2017 vs 2020 ATS indicates Adult Telephone Survey.

The overall 2020 sample included 7129 youths (ages 13-17 years), 3628 young adults (ages 18-20), and 8874 adults (ages ≥21 years). Regardless of interview year, past 30-day substance use across all categories was more prevalent with increasing age through ages 21 to 24 years, then either decreased or remained stable after age 24 years (Table and Figures 1 and 2; eTable 2 in Supplement 1). For instance, in 2020, the prevalence of past 30-day any tobacco use for age groups 13 to 15 years, 16 to 17 years, 18 to 20 years, 21 to 24 years, and 25 years or older was 2.6% (95% CI, 2.1%-3.3%), 9.2% (95% CI, 8.2%-10.4%), 22.8% (95% CI, 21.2%-24.4%), 30.9% (95% CI, 28.5%-33.3%), and 23.1% (95% CI, 22.1%-24.2%), respectively; for past 30-day any alcohol use, 4.6% (95% CI, 3.9%-5.4%), 12.9% (95% CI, 11.7%-14.1%), 35.0% (95% CI, 33.2%-36.8%), 65.2% (95% CI, 62.1%-68.1%), and 53.7% (95% CI, 51.2%-56.0%), respectively; for past 30-day binge drinking, 0.3% (95% CI, 0.1%-0.6%), 2.4% (95% CI, 2.0%-3.0%), 8.6% (95% CI, 7.6%-9.7%), 12.4% (95% CI, 10.8%-14.1%), and 5.9% (95% CI, 5.3%-6.6%), respectively; for past 30-day cannabis use, 1.7% (95% CI, 1.3%-2.2%), 7.6% (95% CI, 6.6%-8.7%), 19.1% (95% CI, 17.4%-20.8%), 27.0% (95% CI, 24.9%-29.3%), and 12.4% (95% CI, 11.4%-13.5%), respectively; and for past 30-day use of other illegal or misused prescription drugs 1.0% (95% CI, 0.7%-1.5%), 1.8% (95% CI, 1.3%-2.4%), 3.0% (95% CI, 2.4%-3.8%), 5.9% (95% CI, 4.9%-7.1%), and 3.7% (95% CI, 3.1%-4.3%), respectively.

**Table.** Tobacco, Alcohol, and Drug Use by Age in the PATH Study Between 2018-2019 and 2020

Substance use in past 30 d	2018-2019 (Wave 5)^a^		2020 (Wave 5.5/PATH-ATS)^b^			*P* value for difference^d^
	No.^c^	Weighted prevalence (95% CI), %	No.^c^	Weighted prevalence (95% CI), %	Prevalence difference vs previous wave (95% CI), percentage point	
**Age 13-15 y**						
Total No.	5864	NA	3550	NA	NA	NA
Any tobacco^e^	424	6.9 (6.3 to 7.7)	95	2.6 (2.1 to 3.3)	−4.3 (−5.1 to −3.6)	<.001
Alcohol	426	7.1 (6.4 to 7.8)	161	4.6 (3.9 to 5.4)	−2.5 (−3.5 to −1.5)	<.001
Binge drinking^f^	51	0.8 (0.6 to 1.1)	8	0.3 (0.1 to 0.6)^g^	−0.5 (−0.8 to −0.3)	.005
Cannabis	325	5.1 (4.3 to 5.9)	66	1.7 (1.3 to 2.2)	−3.4 (−4.1 to −2.6)	<.001
Other illegal and misused prescription drugs^h^	205	3.5 (3.1 to 4.0)	36	1.0 (0.7 to 1.5)	−2.5 (−3.1 to −1.9)	<.001
**Age 16-17 y**						
Total No.	4459	NA	3579	NA	NA	NA
Any tobacco^e^	831	19.5 (18.4 to 20.7)	310	9.2 (8.2 to 10.4)	−10.3 (−11.6 to −9.0)	<.001
Alcohol	762	18.2 (16.9 to 19.7)	423	12.9 (11.7 to 14.1)	−5.4 (−6.9 to −3.9)	<.001
Binge drinking^f^	160	4.0 (3.3 to 4.8)	79	2.4 (2.0 to 3.0)	−1.5 (−2.4 to −0.7)	<.001
Cannabis	667	14.9 (13.9 to 16.0)	271	7.6 (6.6 to 8.7)	−7.3 (−8.8 to −5.8)	<.001
Other illegal and misused prescription drugs^h^	215	4.7 (4.0 to 5.5)	63	1.8 (1.3 to 2.4)	−2.9 (−3.8 to −2.1)	<.001
**Age 18-20 y**						
No.	6046	NA	4193	NA	NA	NA
Any tobacco^e^	2224	37.8 (36.4 to 39.3)	864	22.8 (21.2 to 24.4)	−15.1 (−16.8 to −13.3)	<.001
Alcohol	2009	35.5 (33.9 to 37.2)	1314	35.0 (33.2 to 36.8)	−0.6 (−2.2 to 1.0)	.48
Binge drinking^f^	585	10.7 (9.9 to 11.7)	318	8.6 (7.6 to 9.7)	−2.2 (−3.4 to −0.9)	.001
Cannabis	1484	25.0 (23.7 to 26.3)	743	19.1 (17.4 to 20.8)	−5.9 (−7.7 to −4.2)	<.001
Other illegal and misused prescription drugs^h^	281	4.9 (4.3 to 5.5)	104	3.0 (2.4 to 3.8)	−1.9 (−2.8 to −0.9)	<.001
**Age 21-24 y**						
No.	5309	NA	2178	NA	NA	NA
Any tobacco^e^	2157	39.0 (37.3 to 40.8)	701	30.9 (28.5 to 33.3)	−8.2 (−10.6 to −5.7)	<.001
Alcohol	3122	60.2 (57.4 to 62.9)	1472	65.2 (62.1 to 68.1)	5.0 (2.3 to 7.7)	<.001
Binge drinking^f^	668	12.3 (11.2 to 13.4)	283	12.4 (10.8 to 14.1)	0.1 (−1.7 to 1.8)	.93
Cannabis	1470	26.5 (24.7 to 28.4)	636	27.0 (24.9 to 29.3)	0.5 (−1.6 to 2.6)	.62
Other illegal and misused prescription drugs^h^	318	5.7 (5.0 to 6.5)	131	5.9 (4.9 to 7.1)	0.2 (−0.9 to 1.4)	.69
**Age ≥25 y**						
No.	21 331	NA	6131	NA	NA	NA
Any tobacco^e^	10 088	26.5 (25.8 to 27.2)	2791	23.1 (22.1 to 24.2)	−3.4 (−4.2 to −2.6)	<.001
Alcohol	12 031	53.6 (51.9 to 55.4)	3746	53.7 (51.2 to 56.0)	0.0 (−1.7 to 1.7)	.97
Binge drinking^f^	2029	6.4 (6.0 to 6.8)	592	5.9 (5.3 to 6.6)	−0.5 (−1.2 to 0.2)	.18
Cannabis	4146	11.3 (10.6 to 11.9)	1359	12.4 (11.4 to 13.5)	1.2 (0.3 to 2.0)	.004
Other illegal and misused prescription drugs^h^	1613	5.8 (5.4 to 6.3)	331	3.7 (3.1, to 4.3)	−2.1 (−2.9 to −1.4)	<.001

Abbreviations: NA, not applicable; PATH, Population Assessment of Tobacco and Health; PATH-ATS, PATH Study Adult Telephone Survey.

^a^ Wave 5 data were collected between December 1, 2018, and November 30, 2019, among 10 323 youths and 32 687 adults.

^b^ Youths ages 13 to 17 years and young adults ages 18 to 19 years were interviewed in wave 5.5, whereas adults ages 20 years and older were interviewed in the PATH-ATS. Wave 5.5 data were collected between July 3 and December 31, 2020, among 7129 youths and 3628 adults. PATH-ATS data were collected between September 10 and December 20, 2020, among 8874 adults.

^c^ Unweighted counts.

^d^ From a Rao-Scott χ^2^ test.

^e^ Use of any of the following tobacco products: cigarettes, e-products, traditional cigars, cigarillos, filtered cigars, hookah, pipe, smokeless tobacco, or snus pouches or loose snus (for youths, also includes dissolvable tobacco, bidis, or kreteks). Respondents who indicated that they had always replaced the tobacco in cigars with cannabis were not considered past 30-day tobacco users unless they had used other noncigar tobacco products.

^f^ Defined as 5 or more drinks in 1 day for men and 4 or more drinks for women.

^g^ Estimate should be interpreted with caution because it has low statistical precision. It is based on a denominator sample size of less than 50 or the coefficient of variation of the estimate or its complement was larger than 30%.

^h^ Includes cocaine or crack, stimulants, other illegal drugs, and prescription painkillers, sedatives, tranquilizers, Ritalin, or Adderall used without a prescription or taken only for the experience or the feeling they caused.

From 2016 to 2019, among youths ages 13 to 15 years and 16 to 17 years, the prevalence of any past 30-day tobacco use increased; whereas prevalence of other substances and binge drinking did not consistently increase or decrease (Table and Figure 1; eTable 2 in Supplement 1). Between 2018 to 2019 and 2020, prevalence of all substances sharply decreased. For example, among youth ages 13 to 15 years and 16 to 17 years, past 30-day prevalence of any tobacco use declined from 6.9% to 2.6% (difference, −4.2 percentage points; 95% CI, −5.1 to −3.6 percentage points; *P* < .001) and from 19.5% to 9.2% (difference, −10.3 percentage points; 95% CI, −11.6 to −9.0 percentage points; *P* < .001), respectively; binge drinking declined from 0.8% to 0.3% (difference, −0.5 percentage points; 95% CI, −0.8 to −0.3 percentage points; *P* = .005) and from 4.0% to 2.4% (difference, −1.6 percentage points; 95% CI, −2.4 to −0.7 percentage points; *P* < .001), respectively; cannabis use declined from 5.1% to 1.7% (difference, −3.4 percentage points; 95% CI, −4.1 to −2.6 percentage points; *P* < .001) and from 14.9% to 7.6% (difference, −7.3 percentage points; 95% CI, −8.8 to −5.8 percentage points; *P* < .001), respectively; use of other illegal or misused prescription drugs declined from 3.5% to 1.0% (difference −2.5 percentage points; 95% CI, −3.1 to −1.9 percentage points; *P* < .001) and from 4.7% to 1.8% (difference, −2.9 percentage points; 95% CI, −3.8 to −2.1 percentage points; *P* < .001), respectively.

Among young adults ages 18 to 20 years, between 2016 to 2017 and 2018 to 2019, prevalence of past 30-day tobacco use increased, while past 30-day any alcohol and other substances decreased (Table and Figure 2; eTable 2 in Supplement 1). Between 2018 to 2019 and 2020, other than for past 30-day any alcohol use, past 30-day prevalence of all substances decreased significantly, including declines in past 30-day prevalence of binge drinking from 10.7% to 8.6% (difference −2.2 percentage points; 95% CI, −3.4 to −0.3 percentage points; *P* < .001).

In adults aged 21 to 24 years, past 30-day tobacco use and cannabis use increased between 2016 to 2017 and 2018 to 2019. Comparing 2018 to 2019 with 2020, past 30-day tobacco use declined, and use of any alcohol increased significantly (Table and Figure 2; eTable 2 in Supplement 1). Past 30-day prevalence of any tobacco use declined from 39.0% to 30.9% (difference −8.2 percentage points, 95% CI, −10.6 to −5.7 percentage points; *P* < .001); use of any alcohol increased from 60.2% to 65.2% (difference, 5.0 percentage points; 95% CI, 2.3 to 7.7 percentage points; *P* < .001); binge drinking, cannabis use, and use of other illegal and misused prescription drugs did not vary significantly.

Among adults aged 25 years and older, past 30-day cannabis use increased between 2016 to 2017 and 2018 to 2019 (Table and Figure 2; eTable 2 in Supplement 1). Between 2018 to 2019 and 2020, past 30-day tobacco use decreased, cannabis use increased, and use of other illegal and misused prescription drugs declined. Past 30-day prevalence of any tobacco use declined from 26.5% to 23.1% (difference, −3.4 percentage points; 95% CI, −4.2 to −2.6 percentage points; *P* < .001); cannabis use increased from 11.3% to 12.4% (difference, 1.2 percentage points; 95% CI, 0.3 to 2.0 percentage points; *P* = .004); use of other illegal or misused prescription drugs declined from 5.8% to 3.7% (difference, −2.1 percentage points; 95% CI, −2.9 to −1.4 percentage points; *P* < .001); use of any alcohol and binge drinking did not change significantly.

When stratified by ages 25 to 49 years compared with 50 years or older, sex, race and ethnicity, or household income, some estimates varied, but no consistent trends were identified (eTables 3-11 in Supplement 1). When stratified by postsecondary degree enrollment (eTable 12 in Supplement 1), binge drinking decreased significantly among 18- to 20-year-old enrollees but not for nonenrollees. Among adults ages 21 to 24 years, any alcohol increased significantly for nonenrollees but not for enrollees.

## Discussion

Compared with 2016 to 2019, PATH Study data from 2020 showed marked reductions in substance use among those ages 13 to 20 years; whereas for those ages 21 to 24 years and 25 years or older, patterns were mixed, other than declines in tobacco use among all age groups. Changes in 2020 were generally consistent across sex, race and ethnicity, and household income subgroups but not across age groups. Although our study cannot confirm causation, declines for youths across multiple substances suggest that changes in social factors triggered by the pandemic, such as reduced peer interactions due to limitations on in-person learning and sports or other social gatherings during this time frame^12^ and increases in parental supervision, may have led youths to reduce substance use more than adults.

Few other sources of US national substance use data during the COVID-19 period were available. A summer 2020 follow-up of pre–COVID-19 school-based Monitoring the Future study participants suggested that rates of cannabis and binge drinking were not lower, but nicotine vaping declined.^13^ Because this Monitoring the Future follow-up was conducted only during the summer months in a relatively small sample of 12th-grade students (n = 582), its results were not comparable with the PATH Study. A US Centers for Disease Control and Prevention web-based survey suggested that many adults used substances to cope with COVID-19–related stress, but no corresponding information was provided about possible reductions in substance use.^14^ Most recently, Monitoring the Future data from students queried in January to June 2021 showed broad declines across nearly all substances for 8th, 10th, and 12th grade students (approximately ages 13-14, 15-16, and 17-18 years), results which were consistent with our findings for teenaged participants in the PATH Study, interviewed in July to December 2020.^15^

In addition to broad declines in youth substance use, the PATH Study found reductions in binge drinking for students aged 18 to 20 years enrolled in a postsecondary program, findings that were consistent with a study that found reduced drinking among college students moving home during the pandemic but not among their peers who had been and remained domiciled at home prior to and during the pandemic.^16^ Also, among adults aged 25 years and older, cannabis use increased, perhaps reflecting long-term increases in cannabis among adults in the US.^17^ Increases may also reflect cannabis use to cope with stress and anxiety associated with the COVID-19 pandemic.^18,19^ Declines in tobacco use across all age groups is consistent with prior reports^20^ and may reflect fears that tobacco would exacerbate COVID-19 pulmonary infections at a time when anxiety about the pandemic was at its peak.^21^

### Limitations

This study has limitations. Because of the differences in mode, period of data collection, and sample size, we advise caution while comparing estimates from 2020 to estimates from previous years. Other studies have reported that shifts in data collection mode may not have affected their results,^22^ but no empirical PATH Study data are available regarding these issues. Additionally, the PATH Study is based on a self-report survey and is subject to recall and social-desirability bias. PATH Study results also may underestimate the prevalence of substance use because the study excluded people experiencing homelessness not living in shelters and excluded institutionalized persons (eg, those in jail/prison), populations who often have more substance use and psychiatric conditions than the general population.^23,24^ Another limitation is inclusion of COVID-19 pandemic data only from the latter months of 2020. Trends may have shifted in 2021 (and 2022) and will need to be addressed by newer data from the PATH Study as well as data from other sources that can be used to examine trends over time. Despite these limitations, strengths included the large, nationally representative sample and multiple years of data, which established 2016 to 2019 trends to compare with changes from 2019 to the latter half of 2020.

## Conclusions

In this cross-sectional study of a nationally representative survey of the US population ages 13 years and older before and during the early months of the COVID-19 pandemic, substance use substantially decreased between 2019 and the latter half of 2020 among youths 13 to 20 years of age; whereas consistent changes were not seen in older persons other than declines in tobacco use, and for ages 25 years and older, cannabis use increased. Pandemic-related social disruptions^12^ may have played a role in generally lower rates of overall substance use for US youths in 2020. Changes in the mode and timing of data collection may also have affected participant reporting of substance use; thus, results should be interpreted with caution.

## Notes

Supplement 1. **eTable 1. **Definitions of and Questions Used to Construct Substance Use Measures **eFigure. **Timeline of PATH Study Waves Showing Data Used in These Analyses **eTable 2. **Tobacco, Alcohol, and Drug Use in the PATH Study by Age Between 2016-2017 and 2020 **eTable 3. **Tobacco, Alcohol, and Drug Use Among Adults 25 Years and Older in the PATH Study Between 2016-2017 and 2020 **eTable 4. **Tobacco, Alcohol, and Drug Use by Age Among Women and Girls in the PATH Study Between 2016-2017 and 2020 **eTable 5. **Tobacco, Alcohol, and Drug Use by Age Among Men and Boys in the PATH Study Between 2016-2017 and 2020 **eTable 6. **Tobacco, Alcohol, and Drug Use by Age Among Non-Hispanic White People in the PATH Study Between 2016-2017 and 2020 **eTable 7. **Tobacco, Alcohol, and Drug Use by Age Among Non-Hispanic Black People in the PATH Study Between 2016-2017 and 2020 **eTable 8. **Tobacco, Alcohol, and Drug Use by Age Among Non-Hispanic People of Other Races in the PATH Study Between 2016-2017 and 2020 **eTable 9. **Tobacco, Alcohol, and Drug Use by Age Among Hispanic People in the PATH Study Between 2016-2017 and 2020 **eTable 10. **Tobacco, Alcohol, and Drug Use by Age Among People With a Household Income <$50,000 per Year in the PATH Study Between 2016-2017 and 2020 **eTable 11. **Tobacco, Alcohol, and Drug Use by Age Among People With a Household Income ≥$50,000 per Year in the PATH Study Between 2016-2017 and 2020 **eTable 12.** Tobacco, Alcohol, and Drug Use by Age and Degree Program Enrollment Status in the PATH Study Between 2016-2017 and 2020

Supplement 2.
Data Sharing Statement
