# Supplementary material for: Tobacco, Alcohol, Cannabis, and Other Drug Use in the US Before and During the Early Phase of the COVID-19 Pandemic
Source: JAMA Netw Open. 2023 Jan 31;6(1):e2254566. doi: 10.1001/jamanetworkopen.2022.54566 (PMC9890285; doi:10.1001/jamanetworkopen.2022.54566)
Supplement: Supplement 1. — eTable 1. Definitions of and Questions Used to Construct Substance Use Measures eFigure. Timeline of PATH Study Waves Showing Data Used in These Analyses eTable 2. Tobacco, Alcohol, and Drug Use in the PATH Study by Age Between 2016-2017 and 2020 eTable 3. Tobacco, Alcohol, and Drug Use Among Adults 25 Years and Older in the PATH Study Between 2016-2017 and 2020 eTable 4. Tobacco, Alcohol, and Drug Use by Age Among Women and Girls in the PATH Study Between 2016-2017 and 2020 eTable 5. Tobacco, Alcohol, and Drug Use by Age Among Men and Boys in the PATH Study Between 2016-2017 and 2020 eTable 6. Tobacco, Alcohol, and Drug Use by Age Among Non-Hispanic White People in the PATH Study Between 2016-2017 and 2020 eTable 7. Tobacco, Alcohol, and Drug Use by Age Among Non-Hispanic Black People in the PATH Study Between 2016-2017 and 2020 eTable 8. Tobacco, Alcohol, and Drug Use by Age Among Non-Hispanic People of Other Races in the PATH Study Between 2016-2017 and 2020 eTable 9. Tobacco, Alcohol, and Drug Use by Age Among Hispanic People in the PATH Study Between 2016-2017 and 2020 eTable 10. Tobacco, Alcohol, and Drug Use by Age Among People With a Household Income <$50,000 per Year in the PATH Study Between 2016-2017 and 2020 eTable 11. Tobacco, Alcohol, and Drug Use by Age Among People With a Household Income ≥$50,000 per Year in the PATH Study Between 2016-2017 and 2020 eTable 12. Tobacco, Alcohol, and Drug Use by Age and Degree Program Enrollment Status in the PATH Study Between 2016-2017 and 2020 [file jamanetwopen-e2254566-s001.pdf]

Supplemental Online Content

Compton WM, Flannagan KSJ, Silveira ML, et al. Tobacco, alcohol, cannabis, and other drug use in the US before and during the early phase of the COVID-19 pandemic. *JAMA Netw Open.* 2023;6(1):e2254566. doi:10.1001/jamanetworkopen.2022.54566

eTable 1. Definitions of and Questions Used to Construct Substance Use Measures

eFigure. Timeline of PATH Study Waves Showing Data Used in These Analyses

eTable 2. Tobacco, Alcohol, and Drug Use in the PATH Study by Age Between 2016-2017 and 2020

eTable 3. Tobacco, Alcohol, and Drug Use Among Adults 25 Years and Older in the PATH Study Between 2016-2017 and 2020

eTable 4. Tobacco, Alcohol, and Drug Use by Age Among Women and Girls in the PATH Study Between 2016-2017 and 2020

eTable 5. Tobacco, Alcohol, and Drug Use by Age Among Men and Boys in the PATH Study Between 2016-2017 and 2020

eTable 6. Tobacco, Alcohol, and Drug Use by Age Among Non-Hispanic White People in the PATH Study Between 2016-2017 and 2020

eTable 7. Tobacco, Alcohol, and Drug Use by Age Among Non-Hispanic Black People in the PATH Study Between 2016-2017 and 2020

eTable 8. Tobacco, Alcohol, and Drug Use by Age Among Non-Hispanic People of Other Races in the PATH Study Between 2016-2017 and 2020

eTable 9. Tobacco, Alcohol, and Drug Use by Age Among Hispanic People in the PATH Study Between 2016-2017 and 2020

eTable 10. Tobacco, Alcohol, and Drug Use by Age Among People With a Household Income <$50,000 per Year in the PATH Study Between 2016-2017 and 2020

eTable 11. Tobacco, Alcohol, and Drug Use by Age Among People With a Household Income ≥$50,000 per Year in the PATH Study Between 2016-2017 and 2020

eTable 12. Tobacco, Alcohol, and Drug Use by Age and Degree Program Enrollment Status in the PATH Study Between 2016-2017 and 2020

This supplemental material has been provided by the authors to give readers additional information about their work.

© 2023 Compton WM et al. *JAMA Network Open.*

eTable 1. Definitions of and Questions Used to Construct Substance Use Measures

Substance use measure	Definition of use	Questions used in categorizing youth participants[a]	Questions used in categorizing adult participants[a]
Past 30 day tobacco use	Use within the past 30 days of any of the following tobacco products: cigarettes, e-products, traditional cigars, cigarillos, filtered cigars, hookah, pipe, smokeless tobacco, or snus in either loose or pouched form (for youth, also includes dissolvable tobacco, bidis or kreteks).	"When was the last time you [smoked a cigarette, even one or two puffs/used an electronic nicotine product, even one or two times/smoked a traditional cigar, even one or two puffs/smoked a cigarillo, even one or two puffs/smoked a filtered cigar, even one or two puffs/smoked a pipe, even one or two puffs/smoked tobacco in a hookah, even one or two puffs/used smokeless tobacco, even one or two times/used snus even one or two times]?"<br>• [Earlier today/Not today but sometime in the past 7 days/Not in the past 7 days but sometime in the past 30 days/Not in the past 30 days but sometime in the past 6 months/Not in the past 6 months but sometime in the past year/1 to 4 years ago/5 or more years ago]	"In the past 30 days, have you [smoked a cigarette, even one or two puffs/used an electronic nicotine product, even one or two times (Electronic nicotine products include e-cigarettes, vape pens, personal vaporizers and mods, e-cigars, e-pipes, e-hookahs, and hookah pens.)/smoked a traditional cigar, even one or two puffs/smoked a cigarillo or filtered cigar, even one or two puffs/smoked a pipe filled with tobacco, even one or two puffs/smoked tobacco in a hookah, even one or two puffs/used smokeless tobacco, such as dip, spit, moist snuff, pouches or chewing tobacco, even on or two times/used snus, even one or two times]?"<br>• [Yes/No]
		"Now think about the past 12 months when you smoked the type of cigar shown below. In the past 12 months, how often was any of the tobacco replaced with marijuana?"[b]<br>• [Every time/Most of the time/Sometimes/Rarely/Never]	"When did you last [smoke a cigarette/use an electronic nicotine product/smoke a traditional cigar/smoke a cigarillo/smoke a filtered cigar/smoke a pipe filled with tobacco/smoke tobacco in a hookah/use smokeless tobacco not including snus/use snus]?"<br>• [In the past hour/Sometime today, but more than an hour ago/Yesterday/Day before yesterday/Three or more days ago]
		"In the past 30 days, have you used dissolvable tobacco products, such as Ariva, Stonewall, or Camel Orbs, Sticks or Strips, even one or two times?"<br>• [Yes/No]	"About how long has it been since you last [smoked a cigarette/took a puff from an electronic nicotine product/smoked a traditional cigar/smoked a cigarillo/smoked a filtered cigar/smoked a pipe filled with tobacco/smoked tobacco in a hookah/used smokeless tobacco/used snus]?"<br>• [Numeric answer in days, months, and/or years]

© 2023 Compton WM et al. *JAMA Network Open.*

		"In the past 30 days, which of the following tobacco products have you tried, even one or two times? Choose all that apply."<br>• [Bidis/Kreteks/I have not tried either of these products]	"On how many of the past 30 days did you [smoke cigarettes/use an electronic nicotine product/smoke traditional cigars/smoke cigarillos/smoke filtered cigars/smoke a pipe filled with tobacco/use smokeless tobacco/use snus]?"<br>• [Numeric answer in days]
			"Which of the following choices best describes your hookah smoking?"<br>• [Every day/Weekly/Monthly/Every couple of months/About once a year]
			"Now think about the past 12 months when you smoked the type of cigar shown below. In the past 12 months, how often was any of the tobacco replaced with marijuana?"[b]<br>• [Every time/Most of the time/Sometimes/Rarely/Never]
Past 30 day alcohol use	Any alcohol use within the past 30 days.	"Have you used alcohol in the past 30 days?"<br>• [Yes/No]	Same as youth
Past 30 day binge drinking	Within the past 30 days, drinking 5 or more drinks in one day (for men) or 4 or more drinks in one day (for women).	"Have you used alcohol in the past 30 days?"<br>• [Yes/No]	Same as youth
		"In the past 30 days, on how many days did you have one or more alcoholic drinks? A drink is a can or bottle of beer; a wine cooler or a glass of wine, champagne, or sherry; a shot of liquor or a mixed drink or cocktail."<br>• [Numeric answer in days]	
		"On average, on those days that you drank in the past 30 days, how many alcoholic drinks did you usually have each day? Count a drink as a can or bottle of beer; a wine cooler or a glass of wine, champagne, or sherry; a shot of liquor or a mixed drink or cocktail."<br>• [Numeric answer in drinks]	

© 2023 Compton WM et al. *JAMA Network Open.*

Past 30 day cannabis use[c]	Any cannabis use within the past 30 days.	"Have you used marijuana, hash, THC, grass, pot or weed in the past 30 days?"<br>• [Yes/No]	"Have you used marijuana, hash, THC, grass, pot or weed in the past 30 days?"<br>• [Yes/No]
		"When was the last time you smoked a [traditional cigar/cigarillo/filtered cigar] as a blunt, even one or two puffs?"<br>• [Earlier today/Not today but sometime in the past 7 days/Not in the past 7 days but sometime in the past 30 days/Not in the past 30 days but sometime in the past 6 months/Not in the past 6 months but sometime in the past year]	"When did you last use marijuana, marijuana concentrates, marijuana waxes, THC, or hash oils in an electronic product such as an e-cigarette, vape, mod, personal vaporizer, e-hookah, or hookah pen?"<br>• [In the past hour/Sometime today, but more than an hour ago/Yesterday/Day before yesterday/Three or more days ago]
		"When did you last use marijuana, marijuana concentrates, marijuana waxes, THC, or hash oils in an electronic product such as an e-cigarette, vape, mod, personal vaporizer, e-hookah, or hookah pen?"<br>• [In the past hour/Sometime today, but more than an hour ago/Yesterday/Day before yesterday/Three or more days ago]	
Past 30 day other illegal or prescription drug use	Within the past 30 days, any use of cocaine, crack, illegal stimulants, or other illegal drugs; or use of prescription painkillers, sedatives, tranquilizers, Ritalin, or Adderall without a prescription or taken only for the experience or the feeling they caused.	"In the past 12 months, have you used any of the following prescription drugs that were not prescribed for you or that you took only for the experience or feeling that they caused?"<br>• [Ritalin or Adderall/Painkillers, sedatives, or tranquilizers]	Same as youth
		"Have you used [Ritalin or Adderall/painkillers, sedatives, or tranquilizers/cocaine or crack/stimulants like methamphetamine or speed/other drugs like heroin, inhalants, solvents, or hallucinogens] in the past 30 days?"<br>• [Yes/No]	

[a]Questions included in the table are those that confirm past 30 day substance use. These questions were asked of participants who also answered affirmatively to questions asking if they had ever used a substance or had used it within the past 12 months.
[b]Respondents who indicated that they had always replaced the tobacco with cannabis were not considered past 30 day tobacco users unless they had used other non-cigar tobacco products.
[c]Cannabis use includes blunt use. Although adults were not directly asked when they last smoked a blunt, adults who reported smoking a blunt within the past 12 months were asked about cannabis use in the past 30 days.

© 2023 Compton WM et al. *JAMA Network Open*.

eFigure 1. Timeline of PATH Study Waves Showing Data Used in These Analyses

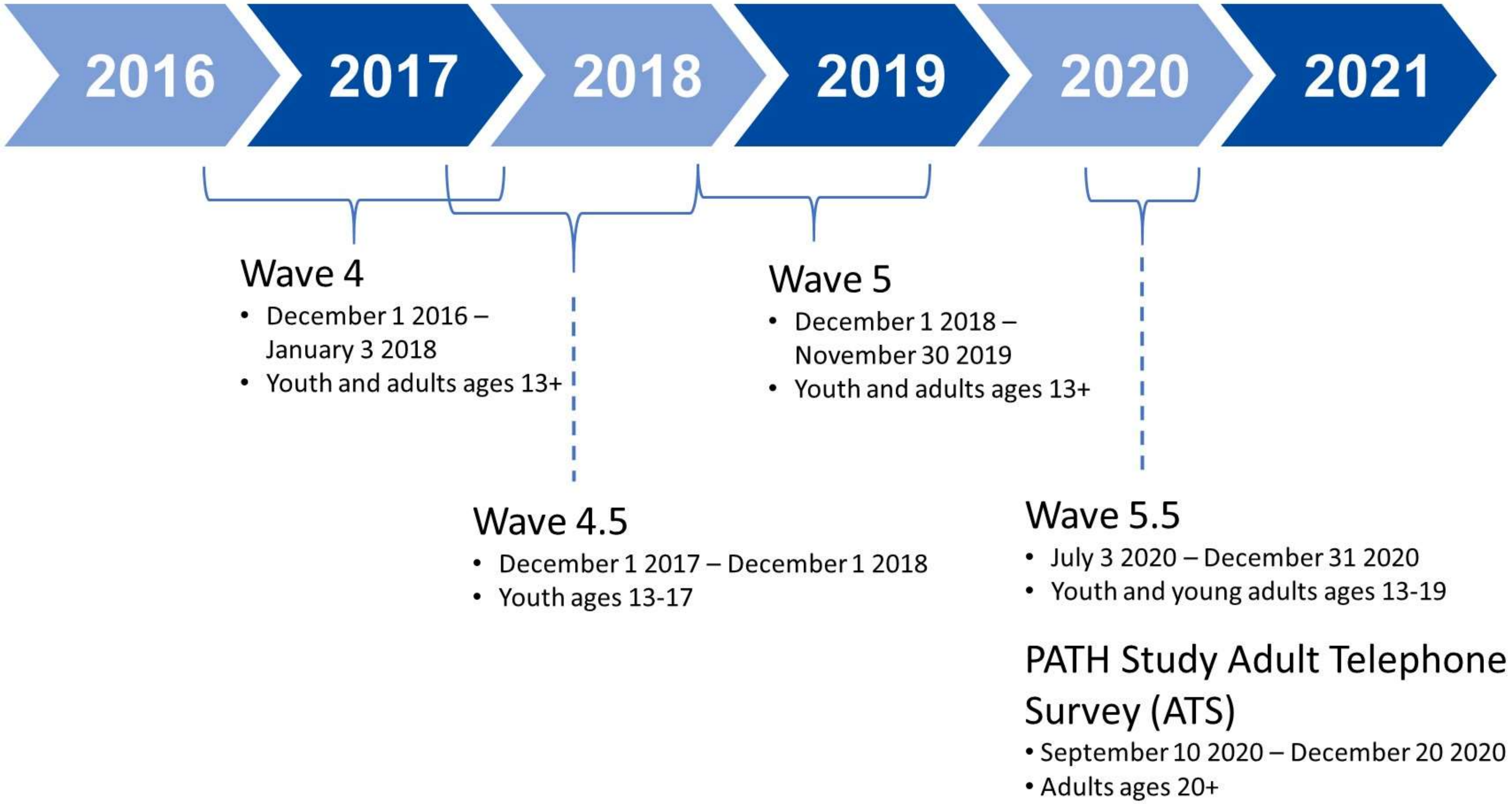

© 2023 Compton WM et al. *JAMA Network Open.*

eTable 2. Tobacco, Alcohol, and Drug Use in the PATH Study by Age Between 2016-2017 and 2020

	2016-2017 (Wave 4)[a]		2017-2018 (Wave 4.5)[b]		2018-2019 (Wave 5)[c]		2020 (Wave 5.5/ATS)[d]	
Substance use in past 30 days	N[e]	Weighted prevalence (95% CI)[f]	N[e]	Weighted prevalence (95% CI)[f]	N[e]	Weighted prevalence (95% CI)[f]	N[e]	Weighted prevalence (95% CI)[f]
Age 13-15 years	7623		6805		5864		3550	
Any tobacco[g]	354	**4.8 (4.3, 5.3)**	429	**6.5 (5.9, 7.1)**	424	**6.9 (6.3, 7.7)**	95	2.6 (2.1, 3.3)
Alcohol	517	**7.1 (6.5, 7.8)**	460	**7.0 (6.4, 7.7)**	426	**7.1 (6.4, 7.8)**	161	4.6 (3.9, 5.4)
Binge drinking[h]	55	**0.8 (0.6, 1.0)**	49	**0.8 (0.6, 1.0)**	51	**0.8 (0.6, 1.1)**	8	0.3 (0.1, 0.6)[j]
Cannabis	330	**4.2 (3.8, 4.8)**	285	**4.1 (3.6, 4.7)**	325	**5.1 (4.3, 5.9)**	66	1.7 (1.3, 2.2)
Other illegal and misused prescription drugs[i]	314	**4.1 (3.6, 4.6)**	298	**4.2 (3.8, 4.8)**	205	**3.5 (3.1, 4.0)**	36	1.0 (0.7, 1.5)
16-17 years	4913		4454		4459		3579	
Any tobacco[g]	691	**14.7 (13.5, 16.0)**	711	**16.7 (15.6, 17.9)**	831	**19.5 (18.4, 20.7)**	310	9.2 (8.2, 10.4)
Alcohol	827	**17.6 (16.1, 19.2)**	729	**17.6 (16.2, 19.0)**	762	**18.2 (16.9, 19.7)**	423	12.9 (11.7, 14.1)
Binge drinking[h]	175	**3.8 (3.2, 4.5)**	154	**3.8 (3.2, 4.5)**	160	**4.0 (3.3, 4.8)**	79	2.4 (2.0, 3.0)
Cannabis	691	**13.8 (12.6, 15.0)**	588	**13.4 (12.5, 14.5)**	667	**14.9 (13.9, 16.0)**	271	7.6 (6.6, 8.7)
Other illegal and misused prescription drugs[i]	261	**5.2 (4.5, 6.1)**	246	**5.3 (4.7, 6.1)**	215	**4.7 (4.0, 5.5)**	63	1.8 (1.3, 2.4)
18-20 years	6212				6046		4193	
Any tobacco[g]	2153	**34.9 (33.3, 36.5)**			2224	**37.8 (36.4, 39.3)**	864	22.8 (21.2, 24.4)
Alcohol	2213	**37.7 (35.7, 39.8)**			2009	35.5 (33.9, 37.2)	1314	35.0 (33.2, 36.8)
Binge drinking[h]	675	**11.6 (10.7, 12.6)**			585	**10.7 (9.9, 11.7)**	318	8.6 (7.6, 9.7)
Cannabis	1475	**23.8 (22.6, 25.0)**			1484	**25.0 (23.7, 26.3)**	743	19.1 (17.4, 20.8)
Other illegal and misused prescription drugs[i]	370	**5.8 (5.2, 6.4)**			281	**4.9 (4.3, 5.5)**	104	3.0 (2.4, 3.8)

© 2023 Compton WM et al. *JAMA Network Open.*

21-24 years	5001				5309		2178	
Any tobacco[g]	2173	**36.8 (34.7, 38.9)**			2157	**39.0 (37.3, 40.8)**	701	30.9 (28.5, 33.3)
Alcohol	3019	**60.0 (56.8, 63.0)**			3122	**60.2 (57.4, 62.9)**	1472	65.2 (62.1, 68.1)
Binge drinking[h]	726	13.1 (12.0, 14.3)			668	12.3 (11.2, 13.4)	283	12.4 (10.8, 14.1)
Cannabis	1326	**23.4 (21.7, 25.3)**			1470	26.5 (24.7, 28.4)	636	27.0 (24.9, 29.3)
Other illegal and misused prescription drugs[i]	363	6.6 (5.8, 7.4)			318	5.7 (5.0, 6.5)	131	5.9 (4.9, 7.1)
25+ years	22425				21331		6131	
Any tobacco[g]	11312	**26.5 (25.9, 27.2)**			10088	**26.5 (25.8, 27.2)**	2791	23.1 (22.1, 24.2)
Alcohol	12870	54.1 (52.4, 55.8)			12031	53.6 (51.9, 55.4)	3746	53.7 (51.2, 56.0)
Binge drinking[h]	2116	6.1 (5.7, 6.5)			2029	6.4 (6.0, 6.8)	592	5.9 (5.3, 6.6)
Cannabis	3768	**9.4 (8.8, 10.0)**			4146	**11.3 (10.6, 11.9)**	1359	12.4 (11.4, 13.5)
Other illegal and misused prescription drugs[i]	1916	**6.3 (5.9, 6.8)**			1613	**5.8 (5.4, 6.3)**	331	3.7 (3.1, 4.3)

Estimates in bold are statistically significantly ($p<0.05$) different from Wave 5.5/ATS according to a Rao-Scott X^2 test.

[a]Wave 4 data were collected between December 1, 2016 and January 3, 2018; n, youth=12,536; n, adults=33,644.

[b]Youth ages 13-17 were interviewed in Wave 4.5 between December 1, 2017 and December 1, 2018; n, youth=11,259.

[c]Wave 5 data were collected between December 1, 2018 and November 30, 2019; n, youth=10,323; n, adults=32,687.

[d]Youth ages 13-17 years and young adults ages 18-19 years were interviewed in Wave 5.5, whereas adults ages 20 years and older were interviewed in the PATH Study Adult Telephone Survey (ATS). Wave 5.5 data were collected between July 3, 2020 and December 31, 2020; n, youth=7,129; n, young adults=3628. ATS data were collected between September 10, 2020 and December 20, 2020; n=8874.

[e]All Ns represent unweighted counts.

[f]Weighted prevalence and 95% Confidence Intervals (CI).

© 2023 Compton WM et al. *JAMA Network Open.*

[g]Use of any of the following tobacco products: cigarettes, e-products, traditional cigars, cigarillos, filtered cigars, hookah, pipe, smokeless tobacco, or snus pouches (for youth, also includes dissolvable tobacco, bidis or kreteks). Respondents who indicated that they had always replaced the tobacco in cigars with cannabis were not considered past 30 day tobacco users unless they had used other non-cigar tobacco products.

[h]Defined as 5 or more drinks in one day for men and 4 or more drinks for women.

[i]Includes cocaine or crack, stimulants, other illegal drugs, and prescription painkillers, sedatives, tranquilizers, Ritalin, or Adderall used without a prescription or taken only for the experience or the feeling they caused.

[j]Estimate should be interpreted with caution because it has low statistical precision. It is based on a denominator sample size of less than 50, or the coefficient of variation of the estimate or its complement is larger than 30 percent.

© 2023 Compton WM et al. *JAMA Network Open.*

eTable 3. Tobacco, Alcohol, and Drug Use Among Adults 25 Years and Older in the PATH Study Between 2016-2017 and 2020

	2016-2017 (Wave 4)[a]		2018-2019 (Wave 5)[b]		2020 (Wave 5.5/ATS)[c]	
Substance use in past 30 days	N[d]	Weighted prevalence (95% CI)[e]	N[d]	Weighted prevalence (95% CI)[e]	N[d]	Weighted prevalence (95% CI)[e]
25-49 years	13382		12851		3269	
Any tobacco[f]	7108	**33.2 (32.2, 34.2)**	6399	**33.7 (32.6, 34.8)**	1619	28.3 (26.7, 29.9)
Alcohol	8200	58.2 (56.1, 60.2)	7687	58.1 (55.9, 60.3)	2198	58.6 (55.1, 62.0)
Binge drinking[g]	1555	8.7 (8.1, 9.4)	1496	9.0 (8.4, 9.7)	413	8.2 (7.3, 9.3)
Cannabis	2869	**13.6 (12.8, 14.4)**	3139	**16.3 (15.4, 17.2)**	973	17.9 (16.5, 19.5)
Other illegal and misused prescription drugs[h]	1204	**6.9 (6.4, 7.6)**	968	**5.8 (5.4, 6.3)**	198	4.1 (3.3, 5.1)
50+ years	9043		8480		2862	
Any tobacco[f]	4204	**20.3 (19.5, 21.2)**	3689	**20.1 (19.2, 21.0)**	1172	18.7 (17.3, 20.2)
Alcohol	4670	50.3 (48.4, 52.2)	4344	49.6 (47.6, 51.6)	1548	49.5 (46.8, 52.1)
Binge drinking[g]	561	3.7 (3.3, 4.2)	533	4.1 (3.7, 4.5)	179	4.0 (3.4, 4.8)
Cannabis	899	**5.5 (4.9, 6.1)**	1007	6.8 (6.1, 7.5)	386	7.7 (6.8, 8.8)
Other illegal and misused prescription drugs[h]	712	**5.7 (5.2, 6.3)**	645	**5.8 (5.2, 6.5)**	133	3.3 (2.6, 4.1)

Estimates in bold are statistically significantly ($p<0.05$) different from Wave 5.5/ATS according to a Rao-Scott X^2 test.

[a]Wave 4 data were collected between December 1, 2016 and January 3, 2018; n, youth=12,536; n, adults=33,644.

[b]Wave 5 data were collected between December 1, 2018 and November 30, 2019; n, youth=10,323; n, adults=32,687.

[c]Youth ages 13-17 years and young adults ages 18-19 years were interviewed in Wave 5.5, whereas adults ages 20 years and older were interviewed in the PATH Study Adult Telephone Survey (ATS). Wave 5.5 data were collected between July 3, 2020 and December 31, 2020; n, youth=7,129; n, young adults=3628. ATS data were collected between September 10, 2020 and December 20, 2020; n=8874.

[d]All Ns represent unweighted counts.

[e]Weighted prevalence and 95% Confidence Intervals (CI).

© 2023 Compton WM et al. *JAMA Network Open.*

[f]Use of any of the following tobacco products: cigarettes, e-products, traditional cigars, cigarillos, filtered cigars, hookah, pipe, smokeless tobacco, or snus pouches (for youth, also includes dissolvable tobacco, bidis or kreteks). Respondents who indicated that they had always replaced the tobacco in cigars with cannabis were not considered past 30 day tobacco users unless they had used other non-cigar tobacco products.

[g]Defined as 5 or more drinks in one day for men and 4 or more drinks for women.

[h]Includes cocaine or crack, stimulants, other illegal drugs, and prescription painkillers, sedatives, tranquilizers, Ritalin, or Adderall used without a prescription or taken only for the experience or the feeling they caused.

© 2023 Compton WM et al. *JAMA Network Open.*

eTable 4. Tobacco, Alcohol, and Drug Use by Age Among Women and Girls in the PATH Study Between 2016-2017 and 2020

	2016-2017 (Wave 4)[a]		2017-2018 (Wave 4.5)[b]		2018-2019 (Wave 5)[c]		2020 (Wave 5.5/ATS)[d]	
Substance use in past 30 days	N[e]	Weighted prevalence (95% CI)[f]	N[e]	Weighted prevalence (95% CI)[f]	N[e]	Weighted prevalence (95% CI)[f]	N[e]	Weighted prevalence (95% CI)[f]
Age 13-15 years	3667		3229		2739		1664	
Any tobacco[g]	151	3.9 (3.3, 4.7)	196	**6.0 (5.1, 7.0)**	201	**6.9 (5.9, 8.0)**	47	2.9 (2.1, 3.9)
Alcohol	303	**8.4 (7.4, 9.5)**	266	**8.4 (7.4, 9.5)**	234	**8.1 (7.0, 9.3)**	90	5.4 (4.3, 6.7)
Binge drinking[h]	31	0.9 (0.6, 1.3)	28	0.9 (0.6, 1.3)	24	0.8 (0.5, 1.2)	4	0.4 (0.1, 1.1)[j]
Cannabis	161	**4.2 (3.5, 5.0)**	137	**4.1 (3.4, 5.0)**	162	**5.3 (4.4, 6.3)**	37	1.9 (1.4, 2.7)
Other illegal and misused prescription drugs[i]	200	**5.1 (4.4, 5.9)**	189	**5.6 (4.8, 6.6)**	136	**4.7 (4.0, 5.6)**	26	1.5 (1.0, 2.3)
16-17 years	2353		2153		2194		1710	
Any tobacco[g]	294	**13.3 (11.8, 15.0)**	337	**16.7 (15.0, 18.5)**	401	**19.0 (17.3, 20.9)**	151	9.3 (7.7, 11.3)
Alcohol	456	**20.0 (18.0, 22.3)**	397	**19.8 (18.1, 21.7)**	442	**21.1 (19.4, 23.0)**	219	13.8 (12.0, 15.8)
Binge drinking[h]	101	**4.4 (3.5, 5.6)**	79	**4.0 (3.2, 4.9)**	89	**4.6 (3.7, 5.8)**	36	2.3 (1.6, 3.2)
Cannabis	327	**13.2 (11.7, 14.8)**	282	**13.3 (12.0, 14.8)**	320	**14.2 (12.6, 16.0)**	118	6.9 (5.6, 8.5)
Other illegal and misused prescription drugs[i]	157	**6.4 (5.4, 7.6)**	132	**5.9 (4.8, 7.1)**	132	**5.8 (4.7, 7.0)**	39	2.3 (1.6, 3.4)
18-20 years	3066				2943		2140	
Any tobacco[g]	891	**29.2 (27.3, 31.1)**			993	**34.1 (32.1, 36.2)**	402	20.5 (18.6, 22.6)
Alcohol	1200	**41.0 (38.3, 43.7)**			1087	38.7 (36.3, 41.1)	708	36.6 (34.2, 39.0)
Binge drinking[h]	329	**11.2 (9.9, 12.6)**			298	**10.9 (9.7, 12.2)**	157	7.9 (6.6, 9.5)
Cannabis	694	**21.9 (20.2, 23.6)**			715	**24.4 (22.7, 26.2)**	378	19.2 (17.2, 21.5)
Other illegal and misused prescription drugs[i]	188	**5.8 (5.0, 6.7)**			161	**5.5 (4.6, 6.6)**	57	3.4 (2.4, 4.8)

© 2023 Compton WM et al. *JAMA Network Open.*

21-24 years	2592				2734		1114	
Any tobacco[g]	938	**29.2 (27.1, 31.4)**			930	**32.4 (30.4, 34.5)**	298	25.0 (22.5, 27.7)
Alcohol	1590	**60.9 (57.3, 64.4)**			1647	**61.6 (58.2, 64.8)**	756	65.9 (61.6, 70.0)
Binge drinking[h]	355	12.1 (10.6, 13.7)			327	11.8 (10.4, 13.3)	137	11.1 (9.1, 13.4)
Cannabis	612	**20.4 (18.6, 22.4)**			680	**23.4 (21.3, 25.6)**	325	26.6 (23.9, 29.5)
Other illegal and misused prescription drugs[i]	186	6.2 (5.3, 7.3)			158	4.9 (4.1, 5.9)	59	4.8 (3.7, 6.3)
25+ years	11448				11103		3184	
Any tobacco[g]	5242	**21.0 (20.2, 21.8)**			4799	**21.2 (20.4, 22.1)**	1360	18.6 (17.3, 20.0)
Alcohol	6114	50.0 (48.1, 51.9)			5933	50.7 (48.8, 52.7)	1834	51.1 (48.0, 54.2)
Binge drinking[h]	872	4.1 (3.7, 4.6)			925	5.2 (4.7, 5.7)	269	4.6 (3.8, 5.6)
Cannabis	1655	**7.2 (6.6, 7.7)**			1944	9.3 (8.8, 9.9)	642	10.2 (9.2, 11.3)
Other illegal and misused prescription drugs[i]	1004	**6.2 (5.6, 6.9)**			851	**5.7 (5.1, 6.3)**	174	3.8 (3.1, 4.8)

Estimates in bold are statistically significantly ($p<0.05$) different from Wave 5.5/ATS according to a Rao-Scott X^2 test.

[a]Wave 4 data were collected between December 1, 2016 and January 3, 2018; n, youth=12,536; n, adults=33,644.

[b]Youth ages 13-17 were interviewed in Wave 4.5 between December 1, 2017 and December 1, 2018; n, youth=11,259.

[c]Wave 5 data were collected between December 1, 2018 and November 30, 2019; n, youth=10,323; n, adults=32,687.

[d]Youth ages 13-17 years and young adults ages 18-19 years were interviewed in Wave 5.5, whereas adults ages 20 years and older were interviewed in the PATH Study Adult Telephone Survey (ATS). Wave 5.5 data were collected between July 3, 2020 and December 31, 2020; n, youth=7,129; n, young adults=3628. ATS data were collected between September 10, 2020 and December 20, 2020; n=8874.

[e]All Ns represent unweighted counts.

[f]Weighted prevalence and 95% Confidence Intervals (CI)

© 2023 Compton WM et al. *JAMA Network Open.*

[g]Use of any of the following tobacco products: cigarettes, e-products, traditional cigars, cigarillos, filtered cigars, hookah, pipe, smokeless tobacco, or snus pouches (for youth, also includes dissolvable tobacco, bidis or kreteks). Respondents who indicated that they had always replaced the tobacco in cigars with cannabis were not considered past 30 day tobacco users unless they had used other non-cigar tobacco products.

[h]Defined as 5 or more drinks in one day for men and 4 or more drinks for women.

[i]Includes cocaine or crack, stimulants, other illegal drugs, and prescription painkillers, sedatives, tranquilizers, Ritalin, or Adderall used without a prescription or taken only for the experience or the feeling they caused.

[j]Estimate should be interpreted with caution because it has low statistical precision. It is based on a denominator sample size of less than 50, or the coefficient of variation of the estimate or its complement is larger than 30 percent.

© 2023 Compton WM et al. *JAMA Network Open.*

eTable 5. Tobacco, Alcohol, and Drug Use by Age Among Men and Boys in the PATH Study Between 2016-2017 and 2020

	2016-2017 (Wave 4)[a]		2017-2018 (Wave 4.5)[b]		2018-2019 (Wave 5)[c]		2020 (Wave 5.5/ATS)[d]		
Substance use in past 30 days	N[e]	Weighted prevalence (95% CI)[f]	N[e]	Weighted prevalence (95% CI)[f]	N[e]	Weighted prevalence (95% CI)[f]	N[e]	Weighted prevalence (95% CI)[f]	P, interaction[g]
Age 13-15 years	3927		3552		3102		1867		
Any tobacco[h]	203	**5.6 (4.9, 6.4)**	230	**6.9 (6.0, 7.9)**	221	**7.0 (6.1, 8.0)**	48	2.4 (1.8, 3.2)	0.33
Alcohol	213	**6.0 (5.2, 6.8)**	194	**5.7 (4.8, 6.8)**	192	**6.2 (5.3, 7.1)**	70	3.9 (3.0, 5.0)	0.77
Binge drinking[i]	24	**0.6 (0.4, 0.9)**	21	**0.7 (0.4, 1.1)**	27	**0.8 (0.6, 1.2)**	4	0.2 (0.1, 0.5)[k]	0.37
Cannabis	168	**4.3 (3.6, 5.1)**	146	**4.0 (3.4, 4.8)**	161	**4.8 (4.1, 5.8)**	29	1.4 (0.9, 2.2)	0.47
Other illegal and misused prescription drugs[j]	113	**3.1 (2.5, 3.8)**	108	**3.0 (2.4, 3.6)**	69	**2.4 (1.8, 3.1)**	10	0.5 (0.3, 0.9)[k]	0.30
16-17 years	2550		2286		2249		1859		
Any tobacco[h]	395	**16.0 (14.4, 17.8)**	373	**16.8 (15.1, 18.8)**	427	**20.0 (18.4, 21.7)**	158	9.2 (7.9, 10.6)	0.56
Alcohol	371	**15.3 (13.5, 17.4)**	330	**15.5 (13.8, 17.5)**	319	**15.5 (13.7, 17.5)**	204	12.1 (10.5, 13.8)	0.11
Binge drinking[i]	74	3.2 (2.5, 4.2)	74	3.6 (2.8, 4.7)	70	3.3 (2.5, 4.4)	43	2.6 (2.0, 3.5)	0.11
Cannabis	362	**14.3 (12.6, 16.2)**	305	**13.6 (12.0, 15.3)**	346	**15.7 (14.1, 17.4)**	152	8.3 (7.1, 9.7)	0.61
Other illegal and misused prescription drugs[j]	103	**4.1 (3.3, 5.1)**	113	**4.8 (4.0, 5.9)**	83	**3.7 (2.9, 4.7)**	24	1.2 (0.8, 2.0)	0.63
18-20 years	3137				3089		2042		
Any tobacco[h]	1260	**40.4 (38.3, 42.6)**			1228	**41.4 (39.6, 43.2)**	459	24.8 (22.5, 27.2)	0.44
Alcohol	1009	34.6 (32.5, 36.7)			922	32.7 (30.8, 34.6)	606	33.6 (31.1, 36.1)	0.16

© 2023 Compton WM et al. *JAMA Network Open.*

Binge drinking[i]	345	**12.0 (10.7, 13.4)**			287	10.6 (9.5, 11.9)	161	9.2 (7.6, 11.1)	0.27
Cannabis	778	**25.6 (23.9, 27.3)**			767	**25.6 (24.0, 27.3)**	365	19.0 (16.9, 21.3)	0.43
Other illegal and misused prescription drugs[j]	182	**5.8 (4.9, 6.9)**			120	**4.3 (3.6, 5.3)**	47	2.6 (1.9, 3.6)	0.94
21-24 years	2404				2566		1058		
Any tobacco[h]	1232	**44.3 (41.3, 47.4)**			1222	**45.5 (43.0, 48.0)**	402	36.4 (33.0, 40.1)	0.90
Alcohol	1427	**59.1 (55.6, 62.4)**			1470	**58.9 (55.8, 61.9)**	712	64.6 (60.8, 68.1)	0.68
Binge drinking[i]	371	14.2 (12.8, 15.8)			339	12.8 (11.3, 14.4)	146	13.7 (11.3, 16.4)	0.46
Cannabis	713	26.5 (24.1, 29.1)			786	29.4 (26.9, 32.0)	308	27.3 (24.5, 30.3)	0.01
Other illegal and misused prescription drugs[j]	177	6.9 (5.8, 8.2)			159	6.4 (5.4, 7.6)	70	6.8 (5.1, 8.8)	0.68
25+ years	10958				10209		2946		
Any tobacco[h]	6065	**32.6 (31.6, 33.6)**			5283	**32.3 (31.2, 33.4)**	1431	28.0 (26.4, 29.7)	0.38
Alcohol	6748	58.6 (56.7, 60.4)			6091	56.9 (54.8, 58.9)	1911	56.4 (53.9, 59.0)	0.58
Binge drinking[i]	1241	8.3 (7.6, 9.0)			1104	7.8 (7.2, 8.4)	322	7.4 (6.5, 8.4)	0.60
Cannabis	2112	**11.8 (11.0, 12.7)**			2200	**13.4 (12.4, 14.3)**	717	14.8 (13.3, 16.5)	0.75

© 2023 Compton WM et al. *JAMA Network Open.*

Other illegal and misused prescription drugs[j]	911	**6.4 (5.8, 7.0)**			761	**6.0 (5.4, 6.6)**	157	3.5 (2.8, 4.4)	0.42

Estimates in bold are statistically ($p<0.05$) significantly different from Wave 5.5/ATS according to a Rao-Scott X^2 test.

[a]Wave 4 data were collected between December 1, 2016 and January 3, 2018; n, youth=12,536; n, adults=33,644.

[b]Youth ages 13-17 were interviewed in Wave 4.5 between December 1, 2017 and December 1, 2018; n, youth=11,259.

[c]Wave 5 data were collected between December 1, 2018 and November 30, 2019; n, youth=10,323; n, adults=32,687.

[d]Youth ages 13-17 years and young adults ages 18-19 years were interviewed in Wave 5.5, whereas adults ages 20 years and older were interviewed in the PATH Study Adult Telephone Survey (ATS). Wave 5.5 data were collected between July 3, 2020 and December 31, 2020; n, youth=7,129; n, young adults=3628. ATS data were collected between September 10, 2020 and December 20, 2020; n=8874.

[e]All Ns represent unweighted counts.

[f]Weighted prevalence and 95% Confidence Intervals (CI)

[g]F test of an interaction term between wave and sex in a logistic regression model with substance use as the outcome and predictors including wave, sex, and their interaction.

[h]Use of any of the following tobacco products: cigarettes, e-products, traditional cigars, cigarillos, filtered cigars, hookah, pipe, smokeless tobacco, or snus pouches (for youth, also includes dissolvable tobacco, bidis or kreteks). Respondents who indicated that they had always replaced the tobacco in cigars with cannabis were not considered past 30 day tobacco users unless they had used other non-cigar tobacco products.

[i]Defined as 5 or more drinks in one day for men and 4 or more drinks for women.

[j]Includes cocaine or crack, stimulants, other illegal drugs, and prescription painkillers, sedatives, tranquilizers, Ritalin, or Adderall used without a prescription or taken only for the experience or the feeling they caused.

[k]Estimate should be interpreted with caution because it has low statistical precision. It is based on a denominator sample size of less than 50, or the coefficient of variation of the estimate or its complement is larger than 30 percent.

© 2023 Compton WM et al. *JAMA Network Open.*

eTable 6. Tobacco, Alcohol, and Drug Use by Age Among Non-Hispanic White People in the PATH Study Between 2016-2017 and 2020

	2016-2017 (Wave 4)[a]		2017-2018 (Wave 4.5)[b]		2018-2019 (Wave 5)[c]		2020 (Wave 5.5/ATS)[d]	
Substance use in past 30 days	N[e]	Weighted prevalence (95% CI)[f]	N[e]	Weighted prevalence (95% CI)[f]	N[e]	Weighted prevalence (95% CI)[f]	N[e]	Weighted prevalence (95% CI)[f]
Age 13-15 years	3326		2966		2604		1667	
Any tobacco[g]	182	**5.5 (4.8, 6.4)**	249	**8.6 (7.6, 9.8)**	238	**8.8 (7.8, 10.0)**	55	3.1 (2.2, 4.4)
Alcohol	279	**8.7 (7.7, 9.7)**	245	**8.6 (7.6, 9.7)**	225	**8.6 (7.5, 9.8)**	101	6.0 (4.8, 7.5)
Binge drinking[h]	32	1.0 (0.7, 1.4)	26	0.9 (0.7, 1.4)	24	0.9 (0.6, 1.3)	6	0.5 (0.2, 1.1)[j]
Cannabis	136	**4.2 (3.5, 5.0)**	127	**4.4 (3.7, 5.3)**	133	**5.0 (4.0, 6.2)**	24	1.3 (0.8, 1.9)
Other illegal and misused prescription drugs[i]	112	**3.5 (2.9, 4.2)**	115	**3.8 (3.2, 4.7)**	78	**3.0 (2.4, 3.8)**	13	0.8 (0.5, 1.6)[j]
16-17 years	2252		2000		1935		1553	
Any tobacco[g]	397	**18.0 (16.0, 20.2)**	407	**20.7 (18.9, 22.6)**	472	**24.8 (23.0, 26.8)**	183	12.4 (10.6, 14.4)
Alcohol	458	**21.6 (19.1, 24.3)**	404	**21.3 (19.0, 23.7)**	419	**22.6 (20.4, 24.8)**	247	17.2 (15.4, 19.1)
Binge drinking[h]	105	**5.0 (4.0, 6.1)**	82	4.4 (3.5, 5.6)	97	**5.4 (4.3, 6.8)**	50	3.5 (2.7, 4.6)
Cannabis	299	**13.5 (11.9, 15.3)**	266	**13.6 (12.2, 15.2)**	300	**15.3 (13.6, 17.2)**	121	8.1 (6.6, 9.8)
Other illegal and misused prescription drugs[i]	108	**5.0 (4.1, 6.0)**	90	**4.5 (3.6, 5.6)**	83	**4.4 (3.4, 5.6)**	28	1.8 (1.2, 2.7)
18-20 years	2949				2710		1876	
Any tobacco[g]	1178	**39.4 (37.1, 41.8)**			1185	**44.5 (42.4, 46.7)**	503	28.7 (26.5, 31.0)
Alcohol	1286	**45.3 (42.5, 48.1)**			1097	42.9 (40.5, 45.3)	735	41.8 (38.9, 44.9)
Binge drinking[h]	428	**14.8 (13.4, 16.3)**			350	**14.0 (12.5, 15.7)**	197	11.0 (9.4, 13.0)
Cannabis	731	**25.0 (23.1, 27.0)**			677	**26.1 (24.1, 28.3)**	372	20.9 (18.5, 23.5)
Other illegal and misused prescription drugs[i]	193	**6.4 (5.6, 7.4)**			137	**5.4 (4.6, 6.4)**	54	3.3 (2.5, 4.3)

© 2023 Compton WM et al. *JAMA Network Open.*

21-24 years	2370				2498		1214	
Any tobacco[g]	1102	**39.4 (35.9, 43.0)**			1118	**42.6 (40.0, 45.3)**	415	35.0 (31.6, 38.6)
Alcohol	1586	**65.2 (59.7, 70.2)**			1668	**66.4 (61.9, 70.6)**	871	69.9 (65.6, 73.8)
Binge drinking[h]	394	14.6 (12.9, 16.4)			360	13.6 (12.1, 15.3)	162	13.8 (11.4, 16.7)
Cannabis	630	23.6 (21.0, 26.5)			705	27.1 (24.5, 30.0)	331	26.2 (23.5, 29.1)
Other illegal and misused prescription drugs[i]	219	8.0 (6.8, 9.4)			165	6.0 (4.9, 7.2)	71	6.0 (4.7, 7.7)
25+ years	13576				12692		4113	
Any tobacco[g]	7008	**27.0 (26.1, 27.9)**			6103	**26.6 (25.7, 27.6)**	1803	23.1 (21.8, 24.4)
Alcohol	8388	**60.0 (57.6, 62.4)**			7732	59.0 (56.6, 61.4)	2554	57.1 (53.9, 60.3)
Binge drinking[h]	1316	6.2 (5.8, 6.7)			1263	**6.8 (6.3, 7.3)**	368	5.9 (5.2, 6.7)
Cannabis	2255	**9.5 (8.8, 10.3)**			2427	**11.1 (10.3, 11.9)**	858	12.3 (11.2, 13.6)
Other illegal and misused prescription drugs[i]	1187	**6.5 (6.0, 7.1)**			966	**6.0 (5.5, 6.6)**	196	3.2 (2.7, 3.9)

Estimates in bold are statistically significantly ($p<0.05$) different from Wave 5.5/ATS according to a Rao-Scott X^2 test.

[a]Wave 4 data were collected between December 1, 2016 and January 3, 2018; n, youth=12,536; n, adults=33,644.

[b]Youth ages 13-17 were interviewed in Wave 4.5 between December 1, 2017 and December 1, 2018; n, youth=11,259.

[c]Wave 5 data were collected between December 1, 2018 and November 30, 2019; n, youth=10,323; n, adults=32,687.

[d]Youth ages 13-17 years and young adults ages 18-19 years were interviewed in Wave 5.5, whereas adults ages 20 years and older were interviewed in the PATH Study Adult Telephone Survey (ATS). Wave 5.5 data were collected between July 3, 2020 and December 31, 2020; n, youth=7,129; n, young adults=3628. ATS data were collected between September 10, 2020 and December 20, 2020; n=8874.

[e]All Ns represent unweighted counts.

[f]Weighted prevalence and 95% Confidence Intervals (CI)

© 2023 Compton WM et al. *JAMA Network Open.*

[g]Use of any of the following tobacco products: cigarettes, e-products, traditional cigars, cigarillos, filtered cigars, hookah, pipe, smokeless tobacco, or snus pouches (for youth, also includes dissolvable tobacco, bidis or kreteks). Respondents who indicated that they had always replaced the tobacco in cigars with cannabis were not considered past 30 day tobacco users unless they had used other non-cigar tobacco products.

[h]Defined as 5 or more drinks in one day for men and 4 or more drinks for women.

[i]Includes cocaine or crack, stimulants, other illegal drugs, and prescription painkillers, sedatives, tranquilizers, Ritalin, or Adderall used without a prescription or taken only for the experience or the feeling they caused.

[j]Estimate should be interpreted with caution because it has low statistical precision. It is based on a denominator sample size of less than 50, or the coefficient of variation of the estimate or its complement is larger than 30 percent.

© 2023 Compton WM et al. *JAMA Network Open.*

eTable 7. Tobacco, Alcohol, and Drug Use by Age Among Non-Hispanic Black People in the PATH Study Between 2016-2017 and 2020

	2016-2017 (Wave 4)[a]		2017-2018 (Wave 4.5)[b]		2018-2019 (Wave 5)[c]		2020 (Wave 5.5/ATS)[d]	
Substance use in past 30 days	N[e]	Weighted prevalence (95% CI)[f]	N[e]	Weighted prevalence (95% CI)[f]	N[e]	Weighted prevalence (95% CI)[f]	N[e]	Weighted prevalence (95% CI)[f]
Age 13-15 years	1009		843		691		368	
Any tobacco[g]	26	2.3 (1.4, 3.7)	22	2.5 (1.6, 3.8)	26	3.3 (2.1, 5.2)	10	2.9 (1.5, 5.4)
Alcohol	37	3.5 (2.5, 5.0)	28	3.4 (2.3, 5.0)	23	2.9 (1.9, 4.4)	9	2.3 (1.2, 4.6)
Binge drinking[h]	NA[j]	NA[j]	NA[j]	NA[j]	4	0.4 (0.1, 1.2)[k]	NA[j]	NA[j]
Cannabis	49	4.3 (3.0, 6.1)	37	4.1 (2.8, 6.1)	39	5.2 (3.6, 7.4)	15	3.5 (2.0, 6.2)
Other illegal and misused prescription drugs[i]	61	**6.0 (4.6, 7.6)**	45	**5.4 (4.1, 7.2)**	36	**5.8 (4.0, 8.3)**	7	1.6 (0.6, 3.9)
16-17 years	623		599		608		434	
Any tobacco[g]	54	**8.9 (6.8, 11.7)**	46	**7.9 (5.6, 11.0)**	69	**10.7 (7.9, 14.4)**	16	3.8 (2.2, 6.5)
Alcohol	47	7.4 (5.6, 9.8)	46	7.9 (6.0, 10.4)	52	**8.5 (6.3, 11.4)**	20	4.7 (2.8, 7.7)
Binge drinking[h]	8	1.4 (0.7, 2.9)[k]	7	1.2 (0.5, 2.8)	7	1.1 (0.5, 2.3)[k]	5	1.0 (0.4, 2.5)[k]
Cannabis	86	**13.2 (10.6, 16.3)**	70	**12.2 (9.5, 15.5)**	90	**14.6 (12.0, 17.7)**	27	6.1 (4.1, 8.9)
Other illegal and misused prescription drugs[i]	39	**6.7 (4.6, 9.7)**	47	**7.1 (5.3, 9.4)**	32	5.0 (3.2, 7.9)	10	2.6 (1.3, 4.8)[k]
18-20 years	890				830		591	
Any tobacco[g]	264	**31.7 (28.5, 35.2)**			248	**30.6 (26.7, 34.9)**	79	13.9 (10.7, 17.9)
Alcohol	190	22.0 (19.4, 24.8)			169	20.9 (18.1, 24.0)	116	23.3 (18.7, 28.6)
Binge drinking[h]	30	3.7 (2.6, 5.3)			31	4.0 (2.8, 5.7)	9	1.8 (0.8, 3.9)[k]
Cannabis	216	**24.5 (21.5, 27.7)**			209	**24.6 (21.8, 27.7)**	87	16.8 (12.8, 21.7)
Other illegal and misused prescription drugs[i]	31	4.0 (2.8, 5.7)			34	3.9 (2.6, 5.9)	11	3.5 (1.7, 7.1)[k]

© 2023 Compton WM et al. *JAMA Network Open.*

21-24 years	811				810		249	
Any tobacco[g]	362	**38.2 (33.9, 42.7)**			295	**35.4 (31.7, 39.3)**	70	23.1 (17.9, 29.3)
Alcohol	359	45.4 (40.9, 49.9)			332	43.2 (39.2, 47.2)	127	48.2 (41.7, 54.8)
Binge drinking[h]	53	7.0 (5.3, 9.1)			39	4.7 (3.4, 6.4)	21	5.9 (3.6, 9.6)
Cannabis	235	26.6 (23.3, 30.2)			230	30.0 (26.1, 34.2)	77	28.3 (22.8, 34.5)
Other illegal and misused prescription drugs[i]	38	4.3 (2.7, 7.0)			36	4.9 (3.5, 6.8)	13	5.0 (2.8, 8.8)
25+ years	3250				3184		757	
Any tobacco[g]	1740	**33.6 (32.0, 35.2)**			1654	**34.4 (32.8, 36.1)**	437	30.8 (27.6, 34.2)
Alcohol	1623	45.6 (43.5, 47.7)			1521	45.2 (42.6, 47.7)	441	47.6 (42.4, 52.9)
Binge drinking[h]	239	5.2 (4.4, 6.1)			217	5.0 (4.2, 6.0)	59	4.1 (2.9, 5.7)
Cannabis	639	**12.7 (11.5, 13.9)**			719	15.5 (14.0, 17.0)	216	18.0 (15.1, 21.3)
Other illegal and misused prescription drugs[i]	274	6.6 (5.6, 7.9)			238	6.3 (5.3, 7.4)	52	5.5 (3.6, 8.4)

Estimates in bold are statistically significantly ($p<0.05$) different from Wave 5.5/ATS according to a Rao-Scott X^2 test.

[a]Wave 4 data were collected between December 1, 2016 and January 3, 2018; n, youth=12,536; n, adults=33,644.

[b]Youth ages 13-17 were interviewed in Wave 4.5 between December 1, 2017 and December 1, 2018; n, youth=11,259.

[c]Wave 5 data were collected between December 1, 2018 and November 30, 2019; n, youth=10,323; n, adults=32,687.

[d]Youth ages 13-17 years and young adults ages 18-19 years were interviewed in Wave 5.5, whereas adults ages 20 years and older were interviewed in the PATH Study Adult Telephone Survey (ATS). Wave 5.5 data were collected between July 3, 2020 and December 31, 2020; n, youth=7,129; n, young adults=3628. ATS data were collected between September 10, 2020 and December 20, 2020; n=8874.

[e]All Ns represent unweighted counts.

[f]Weighted prevalence and 95% Confidence Intervals (CI)

[g]Use of any of the following tobacco products: cigarettes, e-products, traditional cigars, cigarillos, filtered cigars, hookah, pipe, smokeless tobacco, or snus pouches (for youth, also includes dissolvable tobacco, bidis or kreteks). Respondents who indicated that they had always replaced the tobacco in cigars with cannabis were not considered past 30 day tobacco users unless they had used other non-cigar tobacco products.

[h]Defined as 5 or more drinks in one day for men and 4 or more drinks for women.

© 2023 Compton WM et al. *JAMA Network Open.*

[i]Includes cocaine or crack, stimulants, other illegal drugs, and prescription painkillers, sedatives, tranquilizers, Ritalin, or Adderall used without a prescription or taken only for the experience or the feeling they caused.
[j]Results are suppressed due to small sample size.
[k]Estimate should be interpreted with caution because it has low statistical precision. It is based on a denominator sample size of less than 50, or the coefficient of variation of the estimate or its complement is larger than 30 percent.

© 2023 Compton WM et al. *JAMA Network Open.*

eTable 8. Tobacco, Alcohol, and Drug Use by Age Among Non-Hispanic People of Other Races in the PATH Study Between 2016-2017 and 2020

	2016-2017 (Wave 4)[a]		2017-2018 (Wave 4.5)[b]		2018-2019 (Wave 5)[c]		2020 (Wave 5.5/ATS)[d]	
Substance use in past 30 days	N[e]	Weighted prevalence (95% CI)[f]	N[e]	Weighted prevalence (95% CI)[f]	N[e]	Weighted prevalence (95% CI)[f]	N[e]	Weighted prevalence (95% CI)[f]
Age 13-15 years	724		656		565		342	
Any tobacco[g]	37	4.7 (3.3, 6.7)	45	**5.3 (3.7, 7.5)**	38	5.0 (3.3, 7.3)	7	2.3 (1.1, 4.7)
Alcohol	45	6.7 (4.8, 9.2)	49	7.2 (5.4, 9.5)	36	6.1 (4.0, 9.1)	16	5.4 (2.8, 10.1)
Binge drinking[h]	NA[j]	NA[j]	7	0.9 (0.4, 2.1)[k]	NA[j]	NA[j]	NA[j]	NA[j]
Cannabis	22	2.6 (1.7, 4.0)	32	**3.8 (2.5, 5.6)**	28	**3.3 (2.1, 5.1)**	6	1.5 (0.7, 3.5)
Other illegal and misused prescription drugs[i]	29	**3.5 (2.3, 5.3)**	33	**4.3 (3.0, 6.2)**	18	3.0 (1.7, 5.1)	4	1.1 (0.4, 3.0)[k]
16-17 years	444		391		429		368	
Any tobacco[g]	69	13.0 (10.1, 16.4)	70	**14.0 (10.9, 17.8)**	82	**15.8 (12.7, 19.6)**	37	8.5 (6.1, 11.8)
Alcohol	67	14.3 (11.1, 18.2)	75	**19.5 (15.1, 24.8)**	72	**17.1 (13.5, 21.3)**	44	11.2 (7.8, 15.7)
Binge drinking[h]	12	2.0 (1.0, 3.8)[k]	15	3.8 (2.3, 6.2)	14	3.2 (1.8, 5.7)	8	1.9 (0.8, 4.3)[k]
Cannabis	66	11.8 (9.0, 15.3)	54	**12.4 (9.2, 16.6)**	67	**14.2 (11.0, 18.0)**	34	7.8 (5.3, 11.3)
Other illegal and misused prescription drugs[i]	26	**4.8 (3.2, 7.2)**	25	**6.1 (3.7, 9.6)**	21	**4.5 (2.9, 6.9)**	6	1.4 (0.6, 3.2)[k]
18-20 years	556				556		389	
Any tobacco[g]	192	**28.4 (24.3, 32.8)**			205	**30.7 (26.6, 35.2)**	76	18.3 (13.6, 24.1)
Alcohol	190	33.4 (28.1, 39.2)			177	31.2 (26.6, 36.1)	128	35.6 (30.0, 41.6)
Binge drinking[h]	49	8.9 (6.3, 12.6)			54	9.7 (7.2, 12.9)	35	8.6 (5.9, 12.4)
Cannabis	155	24.3 (20.4, 28.7)			159	24.9 (21.2, 29.1)	80	19.0 (14.4, 24.6)
Other illegal and misused prescription drugs[i]	40	5.6 (3.9, 7.8)			21	3.5 (2.1, 5.6)	11	2.5 (1.1, 5.5)[k]

© 2023 Compton WM et al. *JAMA Network Open.*

21-24 years	480				472		213	
Any tobacco[g]	195	27.0 (22.6, 32.0)			191	29.8 (24.7, 35.4)	60	23.2 (16.5, 31.5)
Alcohol	298	61.0 (53.4, 68.1)			298	61.4 (55.4, 67.0)	145	68.7 (58.5, 77.3)
Binge drinking[h]	70	9.9 (7.5, 12.9)			49	8.3 (5.8, 11.8)	21	8.1 (5.0, 12.8)
Cannabis	118	**17.9 (13.9, 22.7)**			142	21.9 (17.3, 27.3)	71	29.2 (21.5, 38.2)
Other illegal and misused prescription drugs[i]	32	4.7 (2.9, 7.4)			28	6.0 (3.8, 9.4)	11	5.5 (2.6, 11.1)[k]
25+ years	1579				1514		406	
Any tobacco[g]	777	**18.7 (16.7, 20.9)**			700	**20.0 (17.7, 22.6)**	164	15.0 (11.8, 18.8)
Alcohol	861	45.2 (41.4, 49.1)			818	45.0 (41.3, 48.8)	225	45.6 (39.6, 51.8)
Binge drinking[h]	129	3.9 (3.0, 5.0)			130	4.1 (3.2, 5.2)	41	4.6 (3.0, 7.1)
Cannabis	283	8.3 (6.6, 10.4)			332	11.0 (9.0, 13.4)	87	9.6 (7.0, 13.0)
Other illegal and misused prescription drugs[i]	128	**4.9 (3.7, 6.6)**			108	**4.4 (3.2, 6.2)**	23	2.4 (1.3, 4.4)

Non-Hispanic Other race includes persons who self-identify as American Indian/Alaska Native, Asian Indian, Chinese, Filipino, Japanese, Korean, Vietnamese, Other Asian, Native Hawaiian, Guamanian or Chamorro, Samoan, Other Pacific Islander, and Multiracial.

Estimates in bold are statistically significantly ($p<0.05$) different from Wave 5.5/ATS according to a Rao-Scott X^2 test.

[a]Wave 4 data were collected between December 1, 2016 and January 3, 2018; n, youth=12,536; n, adults=33,644.

[b]Youth ages 13-17 were interviewed in Wave 4.5 between December 1, 2017 and December 1, 2018; n, youth=11,259.

[c]Wave 5 data were collected between December 1, 2018 and November 30, 2019; n, youth=10,323; n, adults=32,687.

[d]Youth ages 13-17 years and young adults ages 18-19 years were interviewed in Wave 5.5, whereas adults ages 20 years and older were interviewed in the PATH Study Adult Telephone Survey (ATS). Wave 5.5 data were collected between July 3, 2020 and December 31, 2020; n, youth=7,129; n, young adults=3628. ATS data were collected between September 10, 2020 and December 20, 2020; n=8874.

[e]All Ns represent unweighted counts.

[f]Weighted prevalence and 95% Confidence Intervals (CI)

[g]Use of any of the following tobacco products: cigarettes, e-products, traditional cigars, cigarillos, filtered cigars, hookah, pipe, smokeless tobacco, or snus pouches (for youth, also includes dissolvable tobacco, bidis or kreteks). Respondents who indicated that they had always replaced the tobacco in cigars with cannabis were not considered past 30 day tobacco users unless they had used other non-cigar tobacco products.

[h]Defined as 5 or more drinks in one day for men and 4 or more drinks for women.

© 2023 Compton WM et al. *JAMA Network Open.*

[i]Includes cocaine or crack, stimulants, other illegal drugs, and prescription painkillers, sedatives, tranquilizers, Ritalin, or Adderall used without a prescription or taken only for the experience or the feeling they caused.
[j]Results are suppressed due to small sample size.
[k]Estimate should be interpreted with caution because it has low statistical precision. It is based on a denominator sample size of less than 50, or the coefficient of variation of the estimate or its complement is larger than 30 percent.

© 2023 Compton WM et al. *JAMA Network Open.*

eTable 9. Tobacco, Alcohol, and Drug Use by Age Among Hispanic People in the PATH Study Between 2016-2017 and 2020

	2016-2017 (Wave 4)[a]		2017-2018 (Wave 4.5)[b]		2018-2019 (Wave 5)[c]		2020 (Wave 5.5/ATS)[d]		
Substance use in past 30 days	N[e]	Weighted prevalence (95% CI)[f]	N[e]	Weighted prevalence (95% CI)[f]	N[e]	Weighted prevalence (95% CI)[f]	N[e]	Weighted prevalence (95% CI)[f]	P, interaction[g]
Age 13-15 years	2240		1979		1679		972		
Any tobacco[h]	94	**4.5 (3.5, 5.7)**	88	**4.4 (3.5, 5.5)**	104	**6.0 (4.9, 7.4)**	19	1.8 (1.1, 2.8)	0.07
Alcohol	138	**6.2 (5.1, 7.4)**	118	**5.8 (4.8, 7.0)**	124	**6.8 (5.7, 8.1)**	26	2.5 (1.6, 3.9)	0.16
Binge drinking[i]	16	**0.7 (0.4, 1.2)**	13	0.7 (0.4, 1.1)	16	**0.9 (0.5, 1.5)**	NA[l]	NA[l]	NA[l]
Cannabis	106	**4.9 (4.0, 6.0)**	73	**3.5 (2.8, 4.4)**	111	**6.2 (5.0, 7.6)**	17	1.4 (0.9, 2.4)	0.07
Other illegal and misused prescription drugs[j]	100	**4.6 (3.8, 5.6)**	88	**4.3 (3.4, 5.6)**	61	**3.5 (2.7, 4.7)**	8	0.8 (0.4, 1.6)[k]	0.94
16-17 years	1496		1344		1320		1030		
Any tobacco[h]	162	**11.6 (9.9, 13.6)**	171	**14.1 (12.2, 16.3)**	179	**14.3 (12.2, 16.6)**	58	5.8 (4.3, 7.7)	0.52
Alcohol	251	**16.9 (14.8, 19.3)**	191	**14.8 (12.8, 17)**	197	**15.3 (13.1, 17.7)**	96	9.7 (7.9, 11.9)	0.53
Binge drinking[i]	49	**3.6 (2.6, 5.0)**	47	**4.0 (2.9, 5.5)**	36	**2.8 (2.0, 3.8)**	13	1.3 (0.7, 2.3)	0.81
Cannabis	221	**15.2 (13.5, 17.1)**	188	**14.9 (12.8, 17.3)**	185	**14.6 (12.8, 16.7)**	76	7.6 (5.9, 9.7)	0.78
Other illegal and misused prescription drugs[j]	87	**5.6 (4.6, 6.9)**	77	**5.9 (4.7, 7.5)**	65	**5.0 (3.9, 6.3)**	17	1.5 (1.0, 2.5)	0.68
18-20 years	1748				1841		1222		
Any tobacco[h]	496	**28.4 (25.8, 31.1)**			552	**30.6 (28.2, 33.1)**	179	15.7 (12.9, 18.8)	0.19
Alcohol	533	30.7 (28.1, 33.4)			546	30.2 (27.7, 32.7)	306	27.0 (24.0, 30.1)	0.16
Binge drinking[i]	163	9.6 (8.3, 11.1)			147	8.3 (7.1, 9.7)	70	7.0 (5.2, 9.3)	0.64

© 2023 Compton WM et al. *JAMA Network Open.*

Cannabis	360	**20.4 (17.9, 23.1)**			419	**23.2 (20.9, 25.7)**	185	16.7 (14.0, 19.7)	0.61
Other illegal and misused prescription drugs[j]	97	**5.1 (4.1, 6.3)**			87	**5.2 (4.1, 6.5)**	26	2.5 (1.5, 4.3)	0.65
21-24 years	1309				1490		484		
Any tobacco[h]	497	34.1 (31.0, 37.5)			536	**36.1 (32.6, 39.7)**	150	28.9 (25.0, 33.2)	0.47
Alcohol	760	**56.6 (52.7, 60.3)**			807	**54.6 (50.4, 58.9)**	321	63.2 (57.4, 68.7)	0.61
Binge drinking[i]	205	15.1 (12.4, 18.1)			217	15.1 (12.9, 17.7)	77	14.6 (11.3, 18.6)	0.83
Cannabis	334	23.4 (21.0, 26.0)			386	25.0 (22.1, 28.2)	155	27.8 (22.7, 33.6)	0.20
Other illegal and misused prescription drugs[j]	73	5.5 (4.2, 7.1)			89	5.4 (4.2, 6.8)	35	6.5 (4.4, 9.3)	0.91
25+ years	3592				3574		768		
Any tobacco[h]	1549	23.0 (21.6, 24.4)			1438	**23.6 (21.9, 25.4)**	339	20.8 (18.0, 23.8)	0.47
Alcohol	1824	**41.4 (38.8, 44.1)**			1796	**42.9 (39.6, 46.3)**	483	49.0 (44.3, 53.7)	0.02
Binge drinking[i]	393	7.8 (6.5, 9.2)			390	7.4 (6.3, 8.6)	115	8.1 (6.4, 10.3)	0.26
Cannabis	535	**7.5 (6.5, 8.5)**			614	9.6 (8.5, 10.8)	182	10.2 (7.9, 13.1)	0.33
Other illegal and misused prescription drugs[j]	295	6.0 (5.2, 7.0)			266	5.5 (4.8, 6.4)	53	4.8 (3.0, 7.6)	0.09

© 2023 Compton WM et al. *JAMA Network Open*.

Estimates in bold are statistically significantly ($p<0.05$) different from Wave 5.5/ATS according to a Rao-Scott X^2 test.

[a]Wave 4 data were collected between December 1, 2016 and January 3, 2018; n, youth=12,536; n, adults=33,644.

[b]Youth ages 13-17 were interviewed in Wave 4.5 between December 1, 2017 and December 1, 2018; n, youth=11,259.

[c]Wave 5 data were collected between December 1, 2018 and November 30, 2019; n, youth=10,323; n, adults=32,687.

[d]Youth ages 13-17 years and young adults ages 18-19 years were interviewed in Wave 5.5, whereas adults ages 20 years and older were interviewed in the PATH Study Adult Telephone Survey (ATS). Wave 5.5 data were collected between July 3, 2020 and December 31, 2020; n, youth=7,129; n, young adults=3628. ATS data were collected between September 10, 2020 and December 20, 2020; n=8874.

[e]All Ns represent unweighted counts.

[f]Weighted prevalence and 95% Confidence Intervals (CI).

[g]F test of an interaction term between wave and race/ethnicity in a logistic regression model with substance use as the outcome and predictors including wave, race/ethnicity, and their interaction.

[h]Use of any of the following tobacco products: cigarettes, e-products, traditional cigars, cigarillos, filtered cigars, hookah, pipe, smokeless tobacco, or snus pouches (for youth, also includes dissolvable tobacco, bidis or kreteks). Respondents who indicated that they had always replaced the tobacco in cigars with cannabis were not considered past 30 day tobacco users unless they had used other non-cigar tobacco products.

[i]Defined as 5 or more drinks in one day for men and 4 or more drinks for women.

[j]Includes cocaine or crack, stimulants, other illegal drugs, and prescription painkillers, sedatives, tranquilizers, Ritalin, or Adderall used without a prescription or taken only for the experience or the feeling they caused.

[k]Estimate should be interpreted with caution because it has low statistical precision. It is based on a denominator sample size of less than 50, or the coefficient of variation of the estimate or its complement is larger than 30 percent.

[l]Results are suppressed due to small sample size.

© 2023 Compton WM et al. *JAMA Network Open.*

eTable 10. Tobacco, Alcohol, and Drug Use by Age Among People With a Household Income <$50,000 per Year in the PATH Study Between 2016-2017 and 2020

	2016-2017 (Wave 4)[a]		2017-2018 (Wave 4.5)[b]		2018-2019 (Wave 5)[c]		2020 (Wave 5.5/ATS)[d]	
Substance use in past 30 days	N[e]	Weighted prevalence (95% CI)[f]	N[e]	Weighted prevalence (95% CI)[f]	N[e]	Weighted prevalence (95% CI)[f]	N[e]	Weighted prevalence (95% CI)[f]
Age 13-15 years	3611		3000		2553		1372	
Any tobacco[g]	196	**5.8 (5.0, 6.8)**	183	**6.5 (5.6, 7.6)**	204	**8.0 (6.8, 9.5)**	41	2.8 (2.0, 4.0)
Alcohol	210	**6.1 (5.3, 7.1)**	154	**5.2 (4.4, 6.2)**	158	**5.9 (4.9, 7.1)**	32	2.3 (1.6, 3.5)
Binge drinking[h]	26	**0.7 (0.5, 1.1)**	14	0.5 (0.3, 0.8)	24	**0.8 (0.5, 1.3)**	NA[j]	NA[j]
Cannabis	196	**5.6 (4.8, 6.7)**	137	**4.6 (3.8, 5.5)**	182	**6.8 (5.6, 8.2)**	29	1.9 (1.3, 2.8)
Other illegal and misused prescription drugs[i]	184	**5.1 (4.4, 5.9)**	159	**5.3 (4.4, 6.3)**	101	**3.8 (3.0, 4.6)**	17	1.1 (0.7, 1.9)
16-17 years	2272		1978		1951		1406	
Any tobacco[g]	340	**16.0 (14.5, 17.6)**	289	**15.7 (13.8, 17.7)**	353	**19.9 (18.0, 21.8)**	126	9.6 (8.0, 11.5)
Alcohol	311	**13.7 (12.1, 15.5)**	229	**11.6 (10.1, 13.2)**	261	**13.8 (12.0, 15.8)**	120	9.0 (7.4, 10.8)
Binge drinking[h]	62	**2.7 (2.0, 3.6)**	48	**2.4 (1.7, 3.4)**	56	**3.1 (2.2, 4.2)**	19	1.4 (0.9, 2.2)
Cannabis	354	**15.1 (13.6, 16.8)**	281	**14.7 (13.1, 16.5)**	313	**16.5 (14.8, 18.3)**	109	7.8 (6.5, 9.4)
Other illegal and misused prescription drugs[i]	138	**6.0 (5.0, 7.4)**	118	**5.7 (4.7, 7.0)**	111	**5.7 (4.6, 7.0)**	26	1.9 (1.3, 2.9)
18-20 years	3602				3494		1942	
Any tobacco[g]	1337	**37.6 (35.4, 39.7)**			1282	**38.0 (36.1, 39.8)**	397	22.9 (20.6, 25.5)
Alcohol	1108	**31.8 (29.9, 33.8)**			980	**29.4 (27.6, 31.3)**	472	26.6 (24.3, 29.0)
Binge drinking[h]	313	**9.1 (8.1, 10.2)**			258	8.0 (7.0, 9.3)	113	6.5 (5.2, 8.2)
Cannabis	894	**24.7 (23.1, 26.4)**			870	**25.2 (23.8, 26.8)**	326	18.2 (15.8, 20.9)
Other illegal and misused prescription drugs[i]	216	**5.8 (5.1, 6.7)**			164	**4.8 (4.1, 5.6)**	52	3.3 (2.3, 4.7)

© 2023 Compton WM et al. *JAMA Network Open.*

21-24 years	3360				3295		1044	
Any tobacco[g]	1563	**39.2 (36.5, 41.9)**			1405	**41.0 (38.9, 43.1)**	358	32.2 (29.0, 35.5)
Alcohol	1853	**54.5 (50.9, 58.1)**			1741	**53.3 (50.6, 55.9)**	648	59.7 (56.0, 63.2)
Binge drinking[h]	460	12.2 (10.8, 13.7)			387	11.4 (10.1, 12.8)	138	11.8 (9.6, 14.4)
Cannabis	911	**24.1 (21.9, 26.6)**			933	26.8 (24.9, 28.9)	325	28.1 (25.1, 31.3)
Other illegal and misused prescription drugs[i]	247	6.7 (5.7, 7.8)			199	5.8 (4.9, 6.7)	64	5.9 (4.5, 7.8)
25+ years	12693				11514		2777	
Any tobacco[g]	7577	**34.4 (33.5, 35.4)**			6521	**34.5 (33.5, 35.6)**	1623	30.8 (29.1, 32.5)
Alcohol	6112	42.3 (40.7, 43.9)			5277	41.3 (39.7, 42.9)	1419	41.9 (39.0, 45.0)
Binge drinking[h]	1321	7.3 (6.7, 7.9)			1121	6.9 (6.3, 7.5)	302	6.6 (5.6, 7.8)
Cannabis	2567	**11.9 (11.3, 12.6)**			2592	13.6 (12.8, 14.5)	740	14.3 (12.9, 15.9)
Other illegal and misused prescription drugs[i]	1297	**7.8 (7.2, 8.5)**			1081	**7.6 (6.9, 8.3)**	206	5.3 (4.3, 6.5)

Estimates in bold are statistically significantly ($p<0.05$) different from Wave 5.5/ATS according to a Rao-Scott X^2 test.

[a]Wave 4 data were collected between December 1, 2016 and January 3, 2018; n, youth=12,536; n, adults=33,644.

[b]Youth ages 13-17 were interviewed in Wave 4.5 between December 1, 2017 and December 1, 2018; n, youth=11,259.

[c]Wave 5 data were collected between December 1, 2018 and November 30, 2019; n, youth=10,323; n, adults=32,687.

[d]Youth ages 13-17 years and young adults ages 18-19 years were interviewed in Wave 5.5, whereas adults ages 20 years and older were interviewed in the PATH Study Adult Telephone Survey (ATS). Wave 5.5 data were collected between July 3, 2020 and December 31, 2020; n, youth=7,129; n, young adults=3628. ATS data were collected between September 10, 2020 and December 20, 2020; n=8874.

[e]All Ns represent unweighted counts.

[f]Weighted prevalence and 95% Confidence Intervals (CI)

[g]Use of any of the following tobacco products: cigarettes, e-products, traditional cigars, cigarillos, filtered cigars, hookah, pipe, smokeless tobacco, or snus pouches (for youth, also includes dissolvable tobacco, bidis or kreteks). Respondents who indicated that they had always replaced the tobacco in cigars with cannabis were not considered past 30 day tobacco users unless they had used other non-cigar tobacco products.

[h]Defined as 5 or more drinks in one day for men and 4 or more drinks for women.

© 2023 Compton WM et al. *JAMA Network Open.*

[i]Includes cocaine or crack, stimulants, other illegal drugs, and prescription painkillers, sedatives, tranquilizers, Ritalin, or Adderall used without a prescription or taken only for the experience or the feeling they caused.
[j]Results are suppressed due to small sample size.

© 2023 Compton WM et al. *JAMA Network Open.*

eTable 11. Tobacco, Alcohol, and Drug Use by Age Among People With a Household Income ≥$50,000 per Year in the PATH Study Between 2016-2017 and 2020

	2016-2017 (Wave 4)[a]		2017-2018 (Wave 4.5)[b]		2018-2019 (Wave 5)[c]		2020 (Wave 5.5/ATS)[d]		
Substance use in past 30 days	N[e]	Weighted prevalence (95% CI)[f]	N[e]	Weighted prevalence (95% CI)[f]	N[e]	Weighted prevalence (95% CI)[f]	N[e]	Weighted prevalence (95% CI)[f]	P, interaction[g]
Age 13-15 years	3795		3657		3212		2111		
Any tobacco[h]	151	**4.0 (3.4, 4.8)**	238	**6.4 (5.7, 7.3)**	214	**6.2 (5.4, 7.2)**	54	2.6 (1.9, 3.5)	0.49
Alcohol	299	**8.1 (7.2, 9.0)**	301	**8.4 (7.5, 9.4)**	261	**7.9 (7.0, 8.9)**	127	6.1 (5.0, 7.3)	0.02
Binge drinking[i]	29	0.8 (0.6, 1.1)	34	**1.0 (0.7, 1.4)**	26	0.8 (0.5, 1.1)	5	0.3 (0.1, 0.8)[k]	0.63
Cannabis	127	**3.3 (2.7, 3.9)**	140	**3.7 (3.0, 4.4)**	138	**3.9 (3.2, 4.8)**	34	1.5 (1.0, 2.1)	0.30
Other illegal and misused prescription drugs[j]	125	**3.4 (2.8, 4.1)**	137	**3.7 (3.1, 4.3)**	99	**3.3 (2.7, 4.0)**	19	0.9 (0.5, 1.7)[k]	0.93
16-17 years	2485		2342		2403		2072		
Any tobacco[h]	335	**13.9 (12.3, 15.7)**	400	**17.4 (15.8, 19.1)**	456	**19.2 (17.6, 20.9)**	180	9.2 (7.9, 10.7)	0.98
Alcohol	497	**20.8 (18.5, 23.2)**	481	**22.0 (20.0, 24.2)**	488	**21.5 (19.7, 23.4)**	294	15.0 (13.4, 16.8)	0.77
Binge drinking[i]	110	**4.8 (3.9, 5.8)**	101	**4.7 (3.8, 5.8)**	101	**4.6 (3.7, 5.7)**	58	3.0 (2.3, 3.8)	0.24
Cannabis	318	**12.8 (11.3, 14.5)**	285	**12.3 (10.9, 13.9)**	340	**13.9 (12.5, 15.4)**	160	7.7 (6.5, 9.1)	0.22
Other illegal and misused prescription drugs[j]	111	**4.5 (3.6, 5.4)**	123	**5.2 (4.3, 6.2)**	102	**4.1 (3.3, 5.1)**	34	1.6 (1.1, 2.3)	0.60
18-20 years	2112				2134		1941		
Any tobacco[h]	672	**32.1 (29.9, 34.4)**			819	**39.1 (36.5, 41.7)**	414	23.5 (21.2, 25.9)	0.88
Alcohol	980	**48.6 (45.3, 52.0)**			920	45.5 (42.8, 48.1)	778	44.3 (41.6, 47.1)	0.34

© 2023 Compton WM et al. *JAMA Network Open.*

Binge drinking[i]	333	**16.5 (14.6, 18.6)**			303	**15.2 (13.7, 16.9)**	191	11.1 (9.6, 12.8)	0.46
Cannabis	499	**23.9 (22.1, 25.8)**			536	**25.6 (23.5, 27.8)**	375	20.8 (18.6, 23.2)	0.23
Other illegal and misused prescription drugs[j]	128	**5.9 (4.9, 7.0)**			101	**5.2 (4.3, 6.4)**	48	3.0 (2.2, 4.2)	0.59
21-24 years	1407				1777		1088		
Any tobacco[h]	507	31.7 (28.5, 35.0)			659	**35.7 (33.1, 38.4)**	332	30.1 (26.7, 33.8)	0.32
Alcohol	1054	73.8 (70.0, 77.2)			1263	72.5 (68.5, 76.2)	799	71.6 (67.4, 75.4)	0.03
Binge drinking[i]	240	15.7 (13.6, 18.0)			262	14.1 (12.4, 16.0)	141	13.2 (10.8, 16.1)	0.57
Cannabis	356	**22.3 (19.9, 25.0)**			480	25.9 (23.0, 29.0)	300	26.2 (23.3, 29.3)	0.71
Other illegal and misused prescription drugs[j]	112	7.1 (5.9, 8.6)			107	5.5 (4.4, 7.0)	64	5.9 (4.3, 7.9)	0.91
25+ years	8992				9173		3274		
Any tobacco[h]	3400	**18.8 (18.0, 19.7)**			3284	**19.5 (18.6, 20.4)**	1137	17.2 (16.0, 18.6)	0.71
Alcohol	6451	**67.8 (65.2, 70.2)**			6477	**66.7 (64.2, 69.1)**	2287	63.1 (60.0, 66.1)	0.02
Binge drinking[i]	755	5.1 (4.6, 5.7)			872	6.2 (5.7, 6.8)	288	5.6 (4.8, 6.4)	0.58
Cannabis	1145	**7.3 (6.6, 8.0)**			1486	**9.5 (8.7, 10.4)**	612	11.1 (9.9, 12.4)	0.22
Other illegal and misused	588	**5.1 (4.6, 5.7)**			508	**4.2 (3.7, 4.8)**	123	2.5 (1.9, 3.1)	0.03

© 2023 Compton WM et al. *JAMA Network Open.*

prescription drugs[j]									

Estimates in bold are statistically significantly ($p<0.05$) different from Wave 5.5/ATS according to a Rao-Scott X^2 test.
[a]Wave 4 data were collected between December 1, 2016 and January 3, 2018; n, youth=12,536; n, adults=33,644.
[b]Youth ages 13-17 were interviewed in Wave 4.5 between December 1, 2017 and December 1, 2018; n, youth=11,259.
[c]Wave 5 data were collected between December 1, 2018 and November 30, 2019; n, youth=10,323; n, adults=32,687.
[d]Youth ages 13-17 years and young adults ages 18-19 years were interviewed in Wave 5.5, whereas adults ages 20 years and older were interviewed in the PATH Study Adult Telephone Survey (ATS). Wave 5.5 data were collected between July 3, 2020 and December 31, 2020; n, youth=7,129; n, young adults=3628. ATS data were collected between September 10, 2020 and December 20, 2020; n=8874.
[e]All Ns represent unweighted counts.
[f]Weighted prevalence and 95% Confidence Intervals (CI).
[g]F test of an interaction term between wave and income in a logistic regression model with substance use as the outcome and predictors including wave, income, and their interaction.
[h]Use of any of the following tobacco products: cigarettes, e-products, traditional cigars, cigarillos, filtered cigars, hookah, pipe, smokeless tobacco, or snus pouches (for youth, also includes dissolvable tobacco, bidis or kreteks). Respondents who indicated that they had always replaced the tobacco in cigars with cannabis were not considered past 30 day tobacco users unless they had used other non-cigar tobacco products.
[i]Defined as 5 or more drinks in one day for men and 4 or more drinks for women.
[j]Includes cocaine or crack, stimulants, other illegal drugs, and prescription painkillers, sedatives, tranquilizers, Ritalin, or Adderall used without a prescription or taken only for the experience or the feeling they caused.
[k]Estimate should be interpreted with caution because it has low statistical precision. It is based on a denominator sample size of less than 50, or the coefficient of variation of the estimate or its complement is larger than 30 percent.

© 2023 Compton WM et al. *JAMA Network Open.*

eTable 12. Tobacco, Alcohol, and Drug Use by Age and Degree Program Enrollment Status in the PATH Study Between 2016-2017 and 2020

	2016-2017 (Wave 4)[a]		2018-2019 (Wave 5)[b]		2020 (Wave 5.5/ATS)[c]		
Substance use in past 30 days	N[d]	Weighted prevalence (95% CI)[e]	N[d]	Weighted prevalence (95% CI)[e]	N[d]	Weighted prevalence (95% CI)[e]	P, interaction[f]
**Enrolled in a degree program[g]**							
18-20 years	2865		2838		2202		
Any tobacco[h]	839	**29.2 (27.0, 31.4)**	948	**33.9 (31.9, 36.1)**	383	19.4 (17.3, 21.6)	0.44
Alcohol	1319	**48.0 (45.1, 50.9)**	1226	45.0 (42.7, 47.4)	849	42.5 (39.9, 45.1)	0.39
Binge drinking[i]	422	**15.3 (13.7, 17.1)**	386	**14.8 (13.4, 16.4)**	197	10.1 (8.7, 11.7)	0.009
Cannabis	667	**23.5 (22.0, 25.1)**	685	**24.3 (22.6, 26.1)**	380	18.6 (16.6, 20.8)	0.90
Other illegal and misused prescription drugs[j]	173	**5.9 (5.0, 6.9)**	128	**4.7 (3.9, 5.7)**	56	3.1 (2.4, 4.1)	0.56
21-24 years	1547		1753		857		
Any tobacco[h]	489	27.3 (24.0, 30.9)	545	**30.4 (27.8, 33.2)**	223	24.5 (21.1, 28.3)	0.55
Alcohol	1066	67.4 (61.0, 73.2)	1184	67.5 (62.7, 72.0)	601	66.5 (62.2, 70.6)	0.005
Binge drinking[i]	231	13.1 (11.1, 15.4)	245	13.9 (11.8, 16.3)	113	11.7 (9.5, 14.3)	0.12
Cannabis	370	**21.0 (17.9, 24.4)**	443	24.8 (22.1, 27.6)	232	24.8 (21.8, 28.1)	0.74
Other illegal and misused prescription drugs[j]	105	6.3 (5.1, 7.9)	117	6.3 (5.1, 7.8)	45	4.6 (3.3, 6.5)	0.05
**Not enrolled in a degree program**							
18-20 years	3328		3194		1986		
Any tobacco[h]	1309	**40.6 (38.3, 42.8)**	1273	**41.8 (39.8, 43.7)**	480	26.8 (24.2, 29.6)	
Alcohol	889	27.5 (25.6, 29.6)	781	26.1 (24.4, 28.0)	463	25.9 (23.5, 28.4)	
Binge drinking[i]	252	8.0 (7.0, 9.1)	199	6.7 (5.9, 7.6)	119	6.6 (5.3, 8.1)	
Cannabis	806	**24.1 (22.3, 25.9)**	798	**25.8 (24.1, 27.5)**	362	19.6 (17.3, 22.2)	
Other illegal and misused prescription drugs[j]	196	**5.7 (4.8, 6.6)**	153	**5.1 (4.3, 6.0)**	48	2.9 (1.9, 4.3)	

© 2023 Compton WM et al. *JAMA Network Open.*

21-24 years	3438		3543		1321		
Any tobacco[h]	1676	**41.8 (39.7, 44.0)**	1605	**43.3 (41.3, 45.3)**	478	34.4 (31.4, 37.5)	
Alcohol	1949	**56.1 (53.6, 58.6)**	1935	**56.7 (54.2, 59.2)**	871	64.4 (60.9, 67.8)	
Binge drinking[i]	495	13.2 (12.0, 14.5)	422	11.5 (10.4, 12.7)	170	12.7 (10.6, 15.2)	
Cannabis	954	**24.8 (23.0, 26.6)**	1026	27.4 (25.4, 29.6)	404	28.2 (25.6, 31.0)	
Other illegal and misused prescription drugs[j]	257	6.7 (5.8, 7.7)	201	5.4 (4.5, 6.4)	86	6.7 (5.3, 8.2)	

Estimates in bold are statistically significantly ($p<0.05$) different from Wave 5.5/ATS according to a Rao-Scott X^2 test.
[a]Wave 4 data were collected between December 1, 2016 and January 3, 2018; n, youth=12,536; n, adults=33,644.
[b]Wave 5 data were collected between December 1, 2018 and November 30, 2019; n, youth=10,323; n, adults=32,687.
[c]Youth ages 13-17 years and young adults ages 18-19 years were interviewed in Wave 5.5, whereas adults ages 20 years and older were interviewed in the PATH Study Adult Telephone Survey (ATS). Wave 5.5 data were collected between July 3, 2020 and December 31, 2020; n, youth=7,129; n, young adults=3628. ATS data were collected between September 10, 2020 and December 20, 2020; n=8874.
[d]All Ns represent unweighted counts.
[e]Weighted prevalence and 95% Confidence Intervals (CI).
[f]F test of an interaction term between wave and degree program enrollment in a logistic regression model with substance use as the outcome and predictors including wave, enrollment, and their interaction.
[g]Includes technical/vocational, 2-year, 4-year, graduate/professional, and other types of programs.
[h]Use of any of the following tobacco products: cigarettes, e-products, traditional cigars, cigarillos, filtered cigars, hookah, pipe, smokeless tobacco, or snus pouches (for youth, also includes dissolvable tobacco, bidis or kreteks). Respondents who indicated that they had always replaced the tobacco in cigars with cannabis were not considered past 30 day tobacco users unless they had used other non-cigar tobacco products.
[i]Defined as 5 or more drinks in one day for men and 4 or more drinks for women.
[j]Includes cocaine or crack, stimulants, other illegal drugs, and prescription painkillers, sedatives, tranquilizers, Ritalin, or Adderall used without a prescription or taken only for the experience or the feeling they caused.

© 2023 Compton WM et al. *JAMA Network Open.*
