# Supplementary material for: Tobacco, Alcohol, Cannabis, and Other Drug Use in the US Before and During the Early Phase of the COVID-19 Pandemic
Source: JAMA Netw Open. 2023 Jan 31;6(1):e2254566. doi: 10.1001/jamanetworkopen.2022.54566 (PMC9890285; doi:10.1001/jamanetworkopen.2022.54566)
Supplement: Supplement 2. — Data Sharing Statement [file jamanetwopen-e2254566-s002.pdf]

Data Sharing Statement

Compton. Tobacco, Alcohol, Cannabis, and Other Drug Use in the US Before and During the Early Phase of the COVID-19 Pandemic. *JAMA Netw Open*. Published January 31, 2023. doi:10.1001/jamanetworkopen.2022.54566

Data
Data available: Yes
Data types: Deidentified participant data
How to access data: 8. United States Department of Health and Human Services. National Institutes of Health. National Institute on Drug Abuse, and United States Department of Health and Human Services. Food and Drug Administration. Center for Tobacco Products. Population Assessment of Tobacco and Health (PATH) Study [United States] Restricted-Use Files. Inter-university Consortium for Political and Social Research [distributor], 2021-12-01. Available at: https://doi.org/10.3886/Series606.
When available: With publication

Supporting Documents
Document types: None

Additional Information
Who can access the data: researchers whose proposed use of the data has been approved
Types of analyses: any purpose
Mechanisms of data availability: with a signed data access agreement
